# Ligand-induced substrate steering and reshaping of [Ag_2_(H)]^+^ scaffold for selective CO_2_ extrusion from formic acid

**DOI:** 10.1038/ncomms11746

**Published:** 2016-06-06

**Authors:** Athanasios Zavras, George N. Khairallah, Marjan Krstić, Marion Girod, Steven Daly, Rodolphe Antoine, Philippe Maitre, Roger J. Mulder, Stefanie-Ann Alexander, Vlasta Bonačić-Koutecký, Philippe Dugourd, Richard A. J. O'Hair

**Affiliations:** 1 School of Chemistry and Bio21 Molecular Science and Biotechnology Institute, The University of Melbourne, 30 Flemington Road, Parkville, Victoria 3010, Australia; 2 ARC Centre of Excellence for Free Radical Chemistry and Biotechnology, 30 Flemington Road, Parkville, Victoria 3010, Australia; 3 Center of Excellence for Science and Technology – Integration of Mediterranean region (STIM) at Interdisciplinary Center for Advanced Science and Technology (ICAST), University of Split, Meštrovićevo šetalište 45, 21000 Split, Croatia; 4 Institut des Sciences Analytiques, Université de Lyon, Université Lyon 1-CNRS-ENS Lyon, 69100 Villeurbanne, France; 5 Institut Lumière Matière, Université Lyon 1-CNRS, Université de Lyon 69622 Villeurbanne Cedex, France; 6 Laboratoire de Chimie Physique, Bâtiment 349, Université Paris-Sud, CNRS, Université Paris-Saclay, F-91405 Orsay, France; 7 CSIRO Manufacturing, Bayview Avenue, Clayton, Victoria 3168, Australia; 8 Humboldt-Universität Berlin, Institut für Chemie, 12489 Berlin, Germany

## Abstract

Metalloenzymes preorganize the reaction environment to steer substrate(s) along the required reaction coordinate. Here, we show that phosphine ligands selectively facilitate protonation of binuclear silver hydride cations, [LAg_2_(H)]^+^ by optimizing the geometry of the active site. This is a key step in the selective, catalysed extrusion of carbon dioxide from formic acid, HO_2_CH, with important applications (for example, hydrogen storage). Gas-phase ion-molecule reactions, collision-induced dissociation (CID), infrared and ultraviolet action spectroscopy and computational chemistry link structure to reactivity and mechanism. [Ag_2_(H)]^+^ and [Ph_3_PAg_2_(H)]^+^ react with formic acid yielding Lewis adducts, while [(Ph_3_P)_2_Ag_2_(H)]^+^ is unreactive. Using bis(diphenylphosphino)methane (dppm) reshapes the geometry of the binuclear Ag_2_(H)^+^ scaffold, triggering reactivity towards formic acid, to produce [dppmAg_2_(O_2_CH)]^+^ and H_2_. Decarboxylation of [dppmAg_2_(O_2_CH)]^+^ via CID regenerates [dppmAg_2_(H)]^+^. These gas-phase insights inspired variable temperature NMR studies that show CO_2_ and H_2_ production at 70 °C from solutions containing dppm, AgBF_4_, NaO_2_CH and HO_2_CH.

## Abstract (web summary)

Designing catalysts and understanding the influence of ligands for particular transformations remains a highly challenging task. Here, the authors show that bisphosphine ligands can alter the geometry of the active site in silver catalysts, driving protonation and ultimately extrusion of carbon dioxide from formic acid.

## Graphical abstract

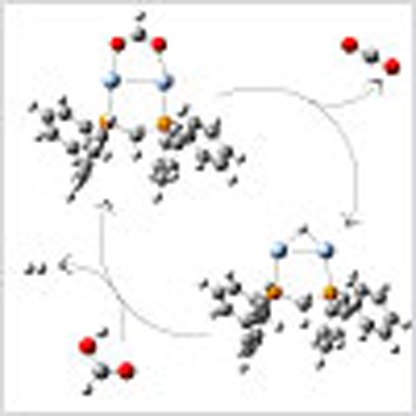

Nature uses a number of design principles to create different classes of enzyme catalysts capable of a wide range of chemical transformations of substrates^1^. A metal ion or metal cluster often has a critical role as a co-factor^2^. A key concept in enzyme catalysis is the preorganization of the reaction environment by the enzyme, directing the substrate to the reaction site, which provides a favourable geometry for the transition state required for bond activation. In essence, the enzyme steers the substrate along the required reaction coordinate to allow the desired transformation to product(s)^3^.

The concept of changing the environment at a metal centre to switch on reactivity has also been recently exploited in gold chemistry. Au(I) complexes prefer to be linear, which is why they are unreactive toward oxidative addition of iodobenzene (Fig. 1a). To promote reactivity, ligand-induced preorganization of the metal centre has been shown to accommodate the geometry of the ensuing oxidative addition of aryl halides (Fig. 1b)^4^. Embedding the metal centre within a ligated nanocluster also facilitates reactivity, which can be further tuned by the choice of ligand (Fig. 1c)^5^.

Here, we use gas-phase experiments and density functional theory (DFT) calculations to examine how the binuclear silver hydride cation, [Ag_2_(H)]^+^ (Fig. 1d,f), can be structurally manipulated by the appropriate choice of phosphine ligands^6^ to switch on the protonation of the hydride by formic acid to liberate hydrogen, which is a key step in the selective, catalysed decomposition of formic acid that does not occur in absence of ligands. We chose [Ag_2_(H)]^+^ since it has been spectroscopically characterized^7^ and ligated variants can readily be formed^8^,^9^,^10^. Formic acid was chosen as a substrate since its decomposition is one of the most widely studied topics in chemistry, with a rich history spanning more than a century^11^,^12^,^13^,^14^. Apart from the academic interest in establishing the mechanism(s) of decomposition, the selective, catalysed decomposition of formic acid has potentially important applications in areas ranging from hydrogen storage^15^,^16^,^17^ through to the generation of *in situ* hydrogenation sources for reduction of organic substrates^18^,^19^.

In the absence of a catalyst, pyrolysis of formic acid proceeds via two primary pathways: decarboxylation (equation (1)) and dehydration (equation (2)). These reactions are coupled by the water–gas shift reaction (Equation (3))^20^,^21^ and have been widely studied experimentally^22^ and theoretically^23^. In the gas-phase, the dehydration channel (Equation (2)) is the dominant reaction^22^, consistent with a lower activation energy, as predicted by DFT calculations^23^.


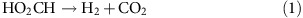



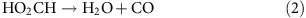



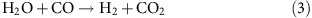


The concept of using metal catalysts to selectively decompose formic acid dates back over 100 years to Sabatier's work on the role of metal and metal oxide catalysts^11^, and the substantial early literature has been reviewed^12^,^13^,^14^. Over the past century, a wide range of metal catalysts have been surveyed for their potential to selectively decarboxylate formic acid (Equation (1)). The types of metal catalysts examined include metal and metal oxide surfaces^14^, mononuclear metal complexes^24^, metal clusters^25^ and metal nanoparticles^26^.

The powerful combination of gas-phase ion–molecule reactions (IMRs), collision-induced dissociation (CID), infrared and ultraviolet action spectroscopy and computational chemistry allows us to examine the role of the ligand (L) in promoting decomposition of formic acid catalysed by the binuclear silver hydride cations, [LAg_2_(H)]^+^. Guided by the right choice of ligand, we have translated our gas-phase results to achieve the selective condensed-phase decarboxylation of formic acid.

## Results

### Catalyst systems

The six systems that we have studied to examine the catalytic cycle (Fig. 2a) for decomposition of formic acid are designated by the letters, which identify the ligands as: **1a**=no ligand; **1b** L=PPh_3_; **1c** L=2 × PPh_3_; **1d** L=bis(diphenylphosphino)methane (dppm); **1e** L=1,2-bis(diphenylphosphino)benzene (dppbz); and **1f** L=bis(diphenylphosphino)ethane (dppe)). DFT calculations reveal that the ligand(s) can induce changes to the geometry of the Ag_2_(H)^+^ scaffold (Fig. 2b).

### Reactions of 1a–1f with formic acid

[Ag_2_(H)]^+^ and its ligated variants, [LAg_2_(H)]^+^, were prepared in the gas-phase via well-established ligand fragmentation reactions (Supplementary Figs 1–6; Supplementary equations 1 and 2), including decarboxylation of coordinated formates, equation (4) (refs 27, 28). The precursor ions were formed via electrospray ionization (ESI).


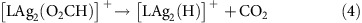


**1a** *m*/*z* 215, reacts via sequential addition of formic acid (Supplementary Fig.7a,b; equations (5) and (6); Fig. 2a (iii) and (v)), as confirmed via mass selection of [Ag_2_(H)(HCOH)]^+^ *m*/*z* 261, and subsequent reaction with formic acid, which yields [Ag_2_(H)(HO_2_CH)_2_]^+^ *m*/*z* 307. CID of [Ag_2_(H)(HCOH)]^+^ regenerates **1a** via loss of formic acid (Fig. 2b and Supplementary Fig. 7c). These results confirm the concept that formic acid is trapped down the wrong reaction pathway (Fig. 2a (iii)).


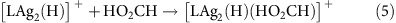



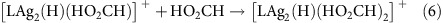


We next tested whether ligation could steer the substrate away from coordination to form a Lewis adduct and toward the hydride site. Blocking one Ag site in **1b** results in the formation of a mono adduct (equation (5), L=Ph_3_P, Supplementary Fig. 8b). Kinetic modelling of the temporal profiles of [(Ph_3_P)_*n*_Ag_2_(H)]^+^ and [(Ph_3_P)_*n*_Ag_2_(H)(HO_2_CH)_2−*n*_]^+^ (Supplementary Table 1) reveals that: (i) the addition of formic acid for *n*=0 and 1 is at ≈1% of the collision rate; (ii) in the case of *n*=0, addition of formic acid is reversible. Blocking both Ag sites makes **1c** unreactive toward formic acid (Supplementary Fig. 8a). By replacing both Ph_3_P ligands with the dppm ligand, the Ag_2_(H)^+^ scaffold of **1e** is compressed, with the P–Ag–H angle deviating substantially away from linearity. As a consequence [dppmAg_2_(H)]^+^ **1d** (*m*/*z* 601) reacts with formic acid to form [dppmAg_2_(O_2_CH)]^+^ and H_2_ (equation (7), Fig. 3a), a reaction that proceeds at ≈ 1% of the collision rate (Supplementary Table 1). When [dppmAg_2_(D)]^+^ *m*/*z* 602, formed via fragmentation of the cluster cation [Ag_3_(μ_3_-D)dppm_3_]^2+^,^8^ was allowed to react with formic acid, the unlabelled formate [dppmAg_2_(O_2_CH)]^+^ and HD are formed (equation (8) and Supplementary Fig. 9b), which is consistent with a mechanism in which the hydride is protonated by formic acid to release H_2_. The reactions given by equations (4) and (7) represent those associated with the catalytic cycle (Fig. 2a) for the selective decarboxylation of formic acid (equation (1)). Indeed, sequential reactions of CID of [dppmAg_2_(O_2_CH)]^+^ to form [dppmAg_2_(H)]^+^ followed by ion-molecule reaction (IMR) with formic acid, allows completion of the cycle multiple times with no significant loss of signal (Supplementary Fig. 9). The other binuclear silver hydride cations, [LAg_2_(H)]^+^, containing bisphosphine ligands also reacted with formic acid to reform the [LAg_2_(O_2_CH)]^+^ and H_2_ (equation (7) and Fig. 3b,c), although the nature of the ligand influences the reaction efficiencies, which follow the order **1d**≈**1e**>>**1f** (Supplementary Table 1).


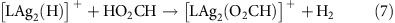



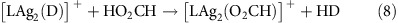


### IR and UV spectroscopy of reactive intermediates

In order to relate the structure of the proposed reactive intermediates **1d** and [dppmAg_2_(O_2_CH)]^+^ to their observed reactivity (Fig. 2a), we next turned our attention to their gas-phase characterization^29^ using infrared multiple photon dissociation (IRMPD) spectroscopy (Fig. 4a,b)^30^ and ultraviolet action-spectroscopy (Fig. 4c,d)^7^,^31^. For each complex, the experimental IRMPD spectrum is compared against the theoretically predicted infrared absorption spectrum of the lowest energy isomer. In the case of [dppmAg_2_(H)]^+^ (*m*/*z* 601), infrared features could only be observed if mass-selected ions were irradiated on resonance with the infrared free electron laser (FEL) and in conjunction with an auxiliary CO_2_ laser^32^. The enhancement of the spectroscopic resolution was such that relatively weak IRMPD features could be observed (Fig. 4a). In the case of [dppmAg_2_(O_2_CH)]^+^ (*m*/*z* 645), however, infrared-induced fragmentation was more easily achieved using the infrared FEL only, and the auxiliary CO_2_ laser was thus not used.

For both [dppmAg_2_(H)]^+^ and [dppmAg_2_(O_2_CH)]^+^, a good match was observed between the experimental and theoretically predicted infrared spectrum for the lowest energy structure. A detailed assignment of the main observed infrared features is provided (Supplementary Table 2). As expected, infrared bands associated with the auxiliary dppm ligand are observed. This is particularly true in the case of the [dppmAg_2_(H)]^+^ spectrum where four bands can be assigned to phenyl in plane ring deformation (998 cm^−1^), CH_2_ twist and P–C_6_H_5_ stretch (1,097 cm^−1^), phenyl in plane CH bending (1,438 cm^−1^), and phenyl CH bending and ring deformation (1,478 cm^−1^).

More importantly, diagnostic bands of the coordination mode of the hydride and formate ligands are also observed. In the case of [dppmAg_2_(H)]^+^ (Fig. 4a), the *μ*^2^ bridging coordination mode of the hydride is well characterized by two bands associated with the asymmetric and symmetric Ag–H stretching bands observed at 900 and 1,250 cm^−1^, respectively, in excellent agreement with the theoretical prediction (916 and 1,236 cm^−1^, respectively). In the case of [dppmAg_2_(O_2_CH)]^+^ (Fig. 4b), it was expected that the positions of the asymmetric and symmetric formate CO stretching bands were sensitive to the formate coordination modes. These two bands are strongly infrared active, and could thus be revealed through IRMPD without the use of the auxiliary CO_2_ laser. As can be seen in Supplementary Table 2, the observed position of these two bands (1,360 and 1,547 cm^−1^, respectively) is in excellent agreement with their predicted positions (1,345 and 1,564 cm^−1^, respectively) for the lowest energy structure of the [dppmAg_2_(O_2_CH)]^+^ complex. It can thus be concluded that IRMPD spectroscopy of [dppmAg_2_(H)]^+^ and [dppmAg_2_(O_2_CH)]^+^, in conjunction with electronic structure calculations, provide clear structural diagnostic of the coordination mode within the Ag_2_H^+^ or Ag_2_(O_2_CH)^+^ scaffolds.

Comparison of the ultravoilet action spectra and calculated time dependent density functional method (TDDFT) spectra using dispersion correction D3 (ref. 33) for the lowest energy structures for [dppmAg_2_(H)]^+^ and [dppmAg_2_(O_2_CH)]^+^ are shown in Fig. 4c,d. Introduction of dispersion correction into TDDFT reduces the distance between two parallel aromatic rings, thus preventing their mobility. In the case of [dppmAg_2_(H)]^+^, the experimental spectrum shows an increase in fragmentation yield as the wavelength decreases, with two superimposed bands at 270 and 235 nm (Fig. 4c). The corresponding TDDFT transitions with dominant oscillator strengths are due to leading excitations from HOMO-1 and HOMO to LUMO+2 (Supplementary Fig. 10), respectively. They involve the Ag_2_H subunit as well as the ligand. In contrast, the S_1_ and S_2_ states located close to 300 nm are characterized by HOMO to LUMO and HOMO-1 to LUMO excitations, respectively, in which Ag_2_ or Ag_2_HP_2_ are more involved than ring subunits of the ligand.

In the case of [dppmAg_2_(O_2_CH)]^+^, a similar action spectrum was obtained. The main difference is a pronounced shoulder at 250 nm resulting from an intense S_1_ transition involving mainly the Ag_2_HP_2_ subunit (Supplementary Fig. 10). The formate has little role in the excitations leading to absorption in this spectral region, which explains the similarity between the two optical spectra. Absorption spectra obtained with TDDFT-D3 are in good agreement with the experimental ultraviolet photodissociation (UVPD) spectra, thus confirming the calculated structural properties. Altogether, on the basis of UVPD and IRMPD spectra, the structural assignments of [dppmAg_2_H]^+^ and [dppmAg_2_(O_2_CH)]^+^ are unambiguous.

### DFT-calculated mechanism of steps associated with the catalytic cycle

**Figure 1:**
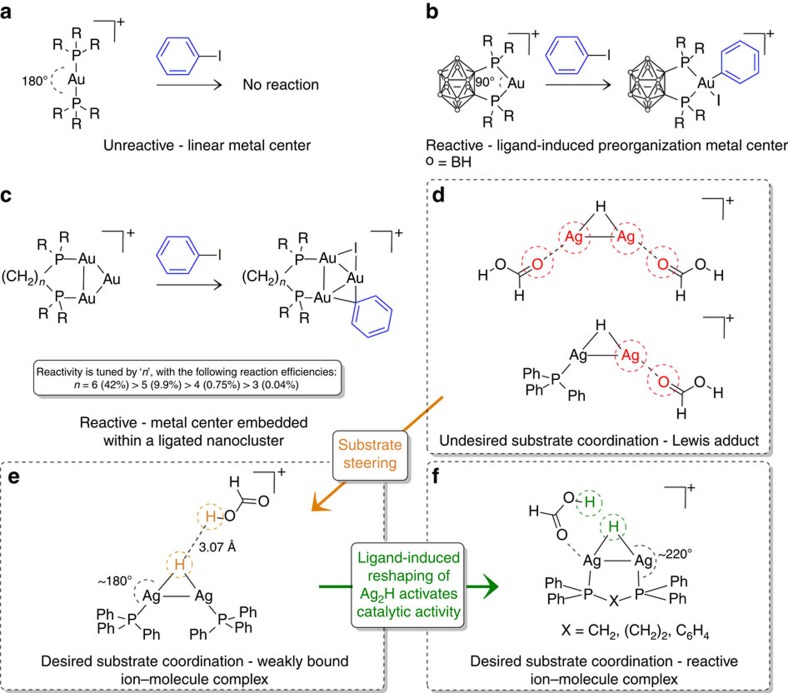
Key concepts for switching on reactivity at coinage metal centres. (**a**) Linear diphosphine Au(I) complexes do not undergo oxidative addition of iodobenzene. Oxidative addition of iodobenzene does occur for: (**b**) bisphosphine Au(I) complexes with P–Au–P bond angles of ≈90°; and (**c**) bisphosphine ligated gold cluster. Switching on desired protonation of binuclear silver hydride cations, [LAg_2_(H)]^+^ by formic acid: (**d**) undesired Lewis adduct formation occurs when silver centres have vacant coordination sites; (**e**) formic acid is steered to active site by phosphine ligands; (**f**) bisphosphine ligands reshape geometry of active site to switch on desired protonation reaction.

**Figure 2:**
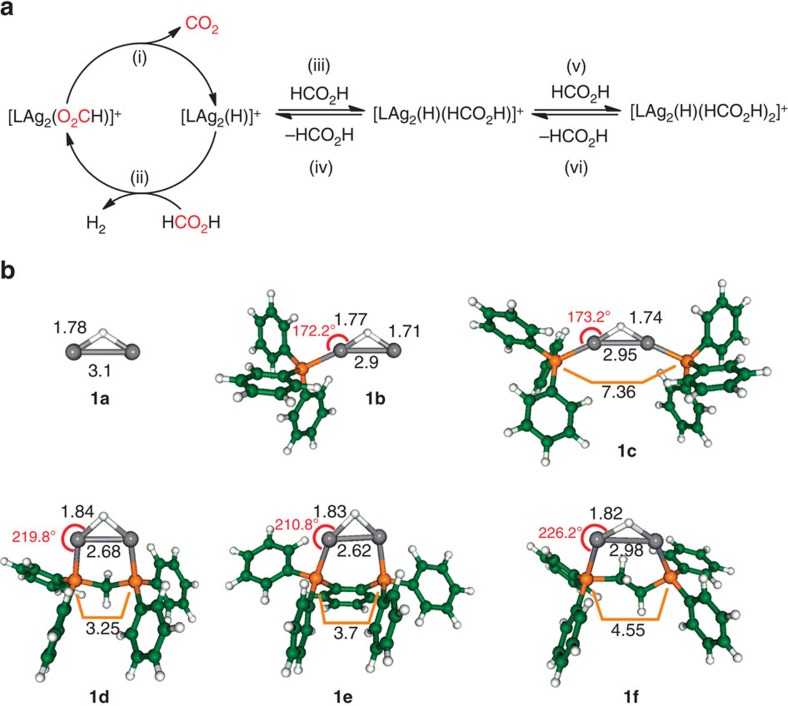
Role of the ligand in the selective decarboxylation of formic acid catalysed by [LAg_2_(H)]^+^. (**a**) catalytic cycle, with following steps: (i), decarboxylation of [LAg_2_(O_2_CH)]^+^ via CID to generate [LAg_2_(H)]^+^. (ii), IMR of [LAg_2_(H)]^+^ to regenerate [LAg_2_(O_2_CH)]^+^. (iii), IMR of [LAg_2_(H)]^+^. (iv), CID of [LAg_2_(H)(HO_2_CH)]^+^ to regenerate [LAg_2_(H)]^+^. (v), IMR of [LAg_2_(H)(HO_2_CH)]^+^ with HO_2_CH to yield [LAg_2_(H)(HO_2_CH)_2_]^+^. (vi), CID of [LAg_2_(H)(HO_2_CH)_2_]^+^ to regenerate [LAg_2_(H)(HO_2_CH)]^+^. Most stable DFT-calculated structures of systems examined: (**b**) **1a**, **1b**, **1c**, **1d**, **1e** and **1f**. DFT calculations used the hybrid functional B3LYP^53^ with def2-TZVP AO basis set^54^ for all atoms and corresponding relativistic effective core potential for Ag atoms^55^. Bond distances are given in Å (black) and P–Ag–H bond angles in degrees (red).

**Figure 3:**
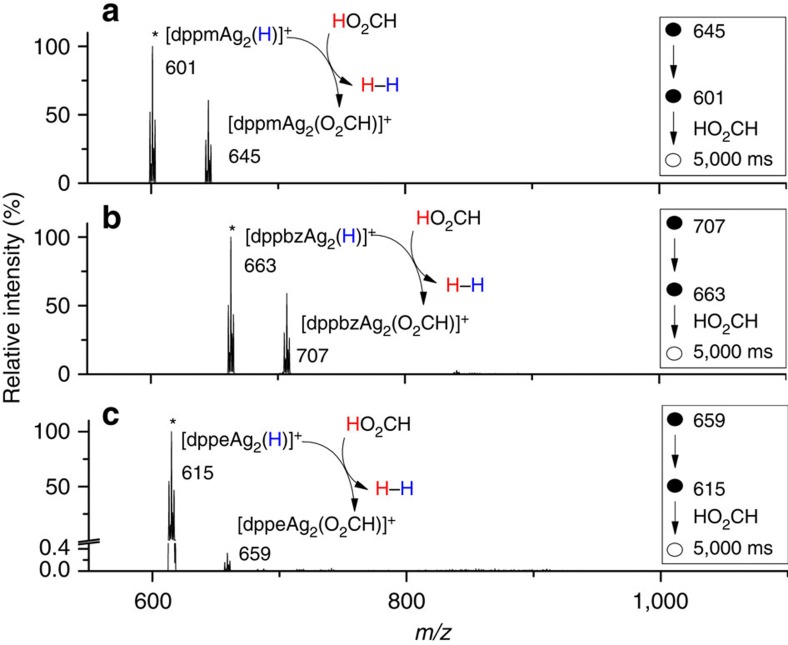
Ion–molecule reaction of formic acid with mass-selected hydrides, [LAg_2_(H)]^+^. (**a**) L= dppm, **1d**, [HO_2_CH]_ion trap_=7.19 × 10^9^ molecules cm^−3^. (**b**) L= dppbz, **1e**, [HO_2_CH]_ion trap_=7.30 × 10^9^ molecules cm^−3^. (**c**) L=dppe, **1f**, [HO_2_CH]_ion trap_=7.09 × 10^9^ molecules cm^−3^. The most intense peak of the isotope cluster is represented by the *m*/*z* value. * Represents the mass selected precursor ion.

**Figure 4:**
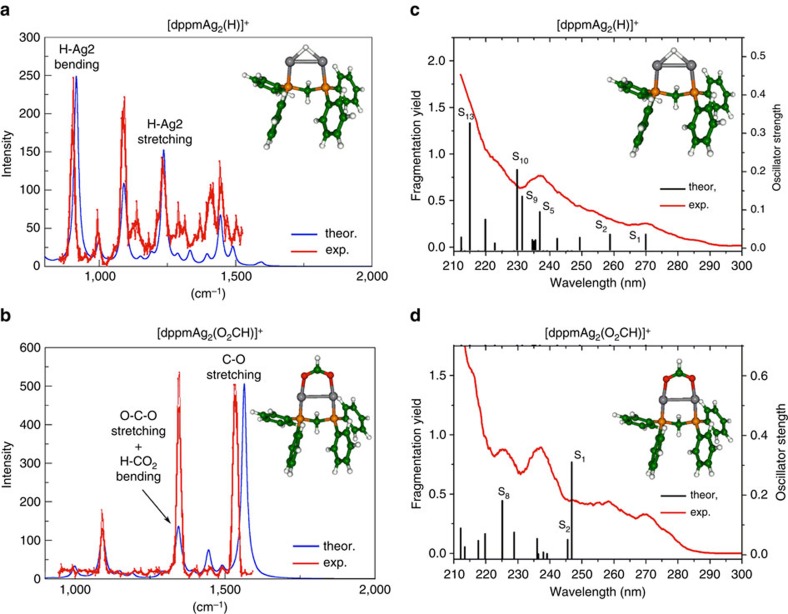
Experimental (red line) and DFT-calculated (blue line) IRMPD and UV spectra (hybrid functional B3LYP with Stuttgart relativistic effective core potential for Ag atoms with corresponding def2-TZVP AO basis set, same AO basis set for all other atoms) of (**a**) [dppmAg_2_(H)]^+^, **1d**. (**b**) [dppmAg_2_(O_2_CH)]^+^. A scaling factor of 0.98 was applied to the calculated harmonic frequencies. UV action spectrum (red line) and calculated TDDFT spectrum with dispersion correction D3 (using CAM-B3LYP functional with Stuttgart relativistic effective core potential for Ag atoms with corresponding def2-TZVP AO basis set for all atoms, the black vertical lines correspond to values of oscillator strength frequency *f*_e_) for the lowest energy structure of: (**c**) [dppmAg_2_(H)]^+^. (**d**) [dppmAg_2_(O_2_CH)]^+^. The analysis of leading excitations is in Supplementary Fig. 10.

**Figure 5:**
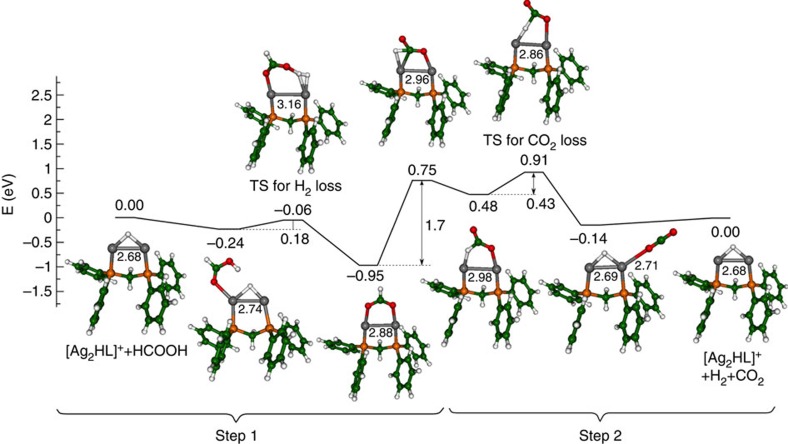
DFT-calculated energy profile for the two reaction steps in the catalytic cycle of Fig. 1a. (Step 1) ion-molecule reaction of formic acid with [dppmAg_2_(H)]^+^; (step 2) CID decarboxylation of [dppmAg_2_(O_2_CH)]^+^. Relative energies are in eV.

The catalytic cycle for the selective decomposition of formic acid involves two distinct types of reactions (Fig. 2a for **1**d, Supplementary Fig. 11 for **1e** and **1f**, Supplementary Table 3 for all systems). The ion–molecule reaction of formic acid with [LAg_2_(H)]^+^ to produce [LAg_2_(O_2_CH)]^+^, and H_2_ must be an exothermic process with barriers that lie below the separated reactants in order for it to occur under the near thermal conditions of the ion-trap^34^. Indeed, DFT calculations reveal this to be the case, for **1d** (Fig. 5 and Supplementary Table 3). The binding energy of cis-formic acid to [dppmAg_2_(H)]^+^ is 0.24 eV, and subsequent reaction via H_2_ formation proceeds via a transition state with barrier of 0.18 eV leading to the formation of [dppmAg_2_(O_2_CH)]^+^, which is exothermic by 0.95 eV. Throughout this reaction, the dppm ligand keeps the Ag_2_ subunit intact, while allowing the Ag–Ag bond length to relax. The overall exothermicities for this first reaction (step 1) for the other [LAg_2_(H)]^+^ complexes examined follow the order L=dppm, (0.95 eV)>L=dppe (0.76 eV)>L=dppbz (0.71 eV)>L=2 × Ph_3_P (0.68 eV)>L=Ph_3_P (0.51 eV), which is in qualitative agreement with experimental findings that dihydrogen release (equation (7)) occurs for L=dppm, dppbz and dppe, but not for the other ligands. The corresponding heights of barriers are **1d** (0.18 eV)<**1f** (0.25 eV)<**1e** (0.36 eV) indicate that all three barriers can overcome under experimental conditions, although the efficiency of the reaction will depend on the height of the barrier. Only in these three cases do the Ag_2_HP_2_ subunits remain intact as can be seen from Fig. 1. Analysis of the charge distributions in these [LAg_2_(H)]^+^ complexes reveals delocalization of positive charge in this subunit (Supplementary Fig. 12).

In contrast, decarboxylation of [LAg_2_(O_2_CH)]^+^ is endothermic as it requires energization through multiple collisions with the helium bath gas during the CID process in order to occur. The mechanism for CO_2_ release involves two steps. First, the formate needs to change from an O,O-bridging ligand to an O-bound ligand. This involves breaking one of the Ag-O bonds via a barrier of 1.7 eV. The next step involves decarboxylation^28^, which proceeds over a barrier of 1.86 eV to release of CO_2_. Altogether, a catalytic cycle involving the selective decomposition of formic acid via the release of H_2_ and CO_2_ (Equation (1)) can occur according to calculated energy profile for [dppmAg_2_(H)]^+^ (Fig. 5), under experimental conditions involving IMR for the spontaneous release of H_2_ and activation via CID for the release of CO_2_. It is to be expected that this is also the case for [dppbzAg_2_(H)]^+^ and [dppeAg_2_(H)]^+^ since that Ag_2_H(X)P_2_ subunit remains intact, although the energetics are slightly less favourable than in the case of [dppmAg_2_(H)]^+^ (Supplementary Table 3).

### Solution-phase selective decarboxylation of formic acid

The fact that [dppmAg_2_(O_2_CH)]^+^ is both readily decarboxylated in the gas-phase and reformed via the reaction of [dppmAg_2_(H)]^+^ with formic acid prompted us to prepare stoichiometrically well-defined solutions in order to use variable temperature ^1^H and ^13^C NMR spectroscopy to examine the evolution of the two gaseous products formed in the selective decarboxylation of formic acid (equation (1)). No H_2_ evolution was observed by ^1^H NMR when a solution of AgBF_4_, ^13^C-labelled formic acid and dppm was heated from 25 to 70 °C (Supplementary Fig. 13). When the experiment was repeated with the addition of sodium formate, no evolution of H_2_ was observed from 25 to 55 °C (Supplementary Fig. 14). When the temperature was raised to 70 °C, H_2_ evolution was observed almost instantly (Supplementary Fig. 15). The evolution of both H_2_ and CO_2_ were observed to increase over time at 70 °C, and both H_2_ and CO_2_ reached a steady-state concentration after ∼11.5 min (Supplementary Figs 15 and 16).

## Discussion

While concepts of steric and electronic effects are well established in guiding the choice of ligand to modulate the reactivity of mononuclear catalysts in homogenous catalysis^35^,^36^, related concepts for choosing ligands to modulate the reactivity of binuclear and cluster catalysts are yet to be fully developed^5^. The value of gas-phase studies employing mass spectrometry (MS)-based methods^37^,^38^ is that they allow a systematic exploration of the factors that control reactivity for all steps in a catalytic cycle^39^,^40^. When used in conjunction with DFT calculations, where mechanistic pathways can be explored, different types of catalysts that vary in the metal, ligand and/or nuclearity that catalyse the same transformation^41^ or which transform the same substrate in different ways^42^ can be directly compared^43^.

Here, we have shown that ligand choice is a crucial factor in designing a binuclear silver hydride cluster that catalyses the selective decarboxylation of formic acid. Ligation of the Ag_2_(H)^+^ scaffold clearly has an influence on its geometry by shortening the Ag–Ag distance and increasing the Ag–H distance (Fig. 1). In the absence of bidentate bridging ligands, the reactivity pattern of **1a**, **1b** and **1c** towards formic acid is consistent with simple Lewis acid/base interactions in which the number of vacant coordination sites in [(Ph_3_P)_*n*_Ag_2_(H)]^+^ dictates how many formic acid molecules can coordinate via the O atom of the C=O to form the adducts [(Ph_3_P)_*n*_Ag_2_(H)(HO_2_CH)_2−*n*_]^+^. Indeed DFT calculations reveal that this coordination mode yields the most stable adducts for *n*=0 and 1, while in the case of *n*=2 only a weakly bound ion–molecule complex is formed (Supplementary Fig. 17) and this is likely to simply dissociate back to separated reactants, which is why no adduct is observed experimentally. In contrast, the tight-bite angles of the bidentate bridging ligands dppm, dppbz and dppe switch on the protonation of the silver hydride in step 1 (Fig. 5) by providing an appropriate geometry to weaken the Ag–H bonds and bend the P–Ag–H away from linearity, thereby allowing coordination of formic acid and subsequent reaction between the coordinated moieties Ag(OCH(O**H**) and Ag(**H**). The ligand further tunes the reactivity as highlighted by both the experimentally determined reaction efficiencies, which follow the order **1d**≈**1e**>>**1f** and the DFT-calculated barrier heights for reaction with cis-formic acid (Supplementary Fig. 18) to release H_2_, which follow the order **1d** (0.18 eV)<**1f** (0. 25 eV)<**1e** (0.36 eV). The ligand also exerts an effect in step 2, with energy resolved CID experiments (Supplementary Fig. 5) providing reactivity orders for the ease of decarboxylation that are in agreement with the DFT-calculated barrier heights: [dppeAg_2_(O_2_CH)]^+^ (1.64 eV)≈[dppbzAg_2_(O_2_CH)]^+^ (1.65 eV)<[dppmAg_2_(O_2_CH)]^+^ (1.86 eV).

Finally, the gas-phase results encouraged us to examine related selective decarboxylation reactions in solution^44^,^45^. We found that both H_2_ and ^13^CO_2_ are evolved when a stoichiometrically well-defined solution containing dppm, AgBF_4_, ^13^C-labelled formic acid and sodium formate was warmed to 70 °C. While the precise nature of the reactive species in solution is unknown, previous studies have shown that: (i) related dppm complexes of silver carboxylates exist as dimers in solution^46^,^47^; (ii) the related silver hydride [(NHC)_2_Ag_2_(H)]^+^ (where, NHC=1,3-bis(2,6-diisopropylphenyl)imidazolin-2-ylidene) exists in solution and reacts with CO_2_ to form [(NHC)_2_Ag_2_(O_2_CH)]^+^, a reaction that is the reverse of decarboxylation of a coordinated formate ligand (equation (4)) studied here^48^.

Two key concepts have emerged from this work: (i) that ligands can have a vital role in reshaping the scaffold of a metal cluster to activate its reactivity towards a substrate; and (ii) that fundamental gas-phase studies can be used to direct the search for new types of metal complexes that promote related reactivity in solution^49^. Together these concepts have allowed us to achieve the selective extrusion of carbon dioxide from formic acid, an important process for applications in hydrogen storage^15^,^16^,^17^.

## Methods

### Materials

Chemicals listed in the Supplementary information were used as received.

### Preparation of silver complexes for MS analysis

*In situ* silver precursor complexes for ESI/MS were typically generated by adding 20 mmol AgX (X=NO_3_^−^ or BF_4_^−^) followed by 10 mmol of phosphine ligand, L (L=PPh_3_, dppm, dppbz or dppe), to 20 ml of freshly prepared solvent mixtures in a 50 ml Quickfit round-bottom flask covered in foil and equipped with a glass stopper and magnetic stir bar. The solution was stirred for at least 5 min and 10 mmol of sodium formate was added. MS experiments were conducted immediately after the addition of sodium formate.

### Gas-phase studies using CID and IMR

Gas-phase experiments on phosphine ligated silver formate clusters were carried out using a Finnigan hybrid linear quadrupole Fourier transform ion-cyclotron resonance mass spectrometer. The silver complexes prepared above were typically diluted in methanol or acetonitrile to a final silver(I) concentration of 50 μM and at least 1.0 ml. The diluted solution was drawn into a 500 μl gas tight borosilicate glass syringe with polytetrafluoroethylene (PTFE) plunger tips and injected into the Finnigan ESI source at a flow rate of 3–5 μl min^−1^. ESI source conditions to yield a stable current of 0.5 μA were: needle potential (3.5–5.0 kV); nitrogen sheath gas pressure (5–10 a.u.). The ion transfer capillary temperature was set to 250 °C. Voltages were: tube lens (≈20.0 V) and capillary voltage (10.0 V). The unimolecular fragmentation/dissociation of mass-selected precursor silver complexes occurred via CID using a normalized collision energy typically between 20 and 25%, and an activation time of 30 ms. IMR were carried by injecting formic acid into the helium bath gas^5^. The stoichiometry of all ions was confirmed by high-resolution MS experiments (Supplementary Table 4).

### Energy-resolved CID experiments

Energy-resolved CID experiments were carried out using a Finnigan 3D ion trap (LCQ) mass spectrometer. The method of Brodbelt was adapted and details are given in the Supplementary methods (see text associated with Supplementary Fig. 4)^50^. The activation voltage was determined by Supplementary equation 3.

### MS for IR action spectroscopy

Infrared spectroscopy of mass-selected ions in the 800–1,600 cm^−1^ range was performed using a 7 Tesla Fourier transform ion cyclotron resonance tandem mass spectrometer (Bruker Apex IV Qe)^51^ equipped with an ESI source and coupled to the infrared FEL beam line of CLIO^52^. This IR FEL delivers ∼10 μs long trains of picosecond pulses at 25 Hz. Ions of interest were accumulated and trapped in a ∼5 cm long hexapole ion-trap pressurized with argon. The trapping delay (∼500 ms) allows for an efficient collisional cooling of the ions. Ions are then pulse extracted to the ICR cell where they are mass-selected, and then irradiated for 1 s. Upon resonant vibrational excitation, dissociation of the selected ion can be monitored via its fragment peaks.

A significant enhancement of the photofragmentation yield can be observed using an auxiliary CO_2_ laser (10 W continuous wave, BFi OPTiLAS, France)^32^. For this purpose, a train of CO_2_ pulses at 25 Hz is generated and synchronized with the IR FEL laser with a retarding delay being on the order of ∼1 μs. This auxiliary CO_2_ laser was used in the case of [dppmAg_2_(H)]^+^ (*m*/*z* 601), and the CO_2_ laser pulse length (25 ms) was adjusted to avoid CO_2_ induced dissociation.

The experimental IRMPD bandwidth (fwhm) for both [dppmAg_2_(H)]^+^ and [dppmAg_2_(O_2_CH)]^+^ is on the order of 20 cm^−1^, as generally observed for IRMPD spectra of other systems obtained using the CLIO FEL.

### MS for ultravoilet action spectroscopy

For ultravoilet action spectroscopy, a dual linear ion trap (LTQ-VELOS, ThermoScientific) was used to generate, mass select and trap ions in a first, high-pressure ion trap, for a controlled duration. During ion trapping, ions can be activated and fragmented by photons or CID. Fragment ions are transmitted to a second ion trap, with low pressure, where they are mass analyzed. A fused-silica window is positioned at the back end of the instrument allowing for the introduction of laser beams in the ultravoilet-visible range along the ion trap axis. Ultravoilet light was generated by doubling the output of an optical parametric oscillator (Horizon optical parametric oscillator pumped by the third harmonic of a Surelite II Nd:YAG laser, Continuum). A mechanical shutter, synchronized with the mass spectrometer, is used to stop the beam at all times except the ‘ion activation window'—that is the time after ion accumulation and before the mass analysis. A single laser pulse was used for the irradiation of the trapped ions and when irradiating ions the normalized collision energy is kept at zero. The fragmentation yield (FY) is given by equation (9).


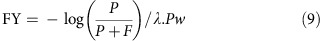


*P* and *F* are the intensities on the mass spectrum for respectively the parent ion and the ensemble of photo-fragment ions. *λ* and *Pw* are respectively the wavelength and measured average power of the incoming ultravoilet laser beam.

### DFT calculations

The extensive search for lowest energy structures and transitions states were performed by the hybrid B3LYP^53^ functional with def2-TZVP atomic basis set^54^, which has been used for all atoms. Silver atoms have been treated by Stuttgart relativistic effective core potential with corresponding atomic orbital (AO) basis set^55^. The same combination of functional and basis set was used for calculation of the infrared spectra, which were scaled by 0.98 to match experimental data. The IR spectrum for neutral monomeric cis-formic acid calculated at this level of DFT theory is also in good agreement with experimental data (Supplementary Fig. 19). For calculations of the absorption spectra TDDFT with the long-range corrected version of the hybrid B3LYP functional, the Coulomb-attenuated CAM-B3LYP functional and TZVP AO basis set has been employed.

Potential interactions between the aromatic rings of dppm, raises the question of whether the dispersion correction within DFT are required. We have tested the influence of dispersion correction on the structural and spectroscopic properties of [dppmAg_2_H]^+^ and [dppmAg_2_(O_2_CH)]^+^ complexes by introducing D3 into DFT and TDDFT^33^. Comparison of the measured and calculated infrared spectra suggest that dispersion corrections has no influence. In contrast, the absorption transitions calculated using the D3 correction in which aromatic rings are involved are only slightly blue shifted, thus improving agreement with the experimental UVPD spectra. Finally, the energy profile shown in Fig. 5 is almost unchanged when single point B3LYP-D3 energy calculations are carried out for each reaction step of the catalytic cycle (Supplementary Table 3).

### NMR spectroscopy experiments

The NMR experiments were performed on a Bruker Avance Av500 NMR spectrometer (500.13 MHz ^1^H frequency) equipped with a 5 mm triple resonance CryoProbe Prodigy probe (^1^H/^19^F–^2^H/^13^C/^15^N). Solutions for analysis by NMR were prepared by dissolving: (1) solution A: AgBF_4_ (195 mg, 1 mmol), dppm (192 mg, 0.5 mmol), ^13^C formic acid (24 mg, 0.5 mmol) and sodium formate (34 mg, 0.5 mmol) in 1 ml of deuteroacetonitrile; (2) solution B: same as solution A, but without sodium formate added. NMR experiments were performed with the sample held at temperatures between +25 °C and +70 °C (±0.1 °C). Chemical shifts for ^1^H experiments are referenced to the residual protonated solvent signal (CD_2_HCN, *δ* 1.94 ppm); ^13^C referenced to the solvent signal (CD_3_CN, *δ* 1.39 ppm). One-dimensional NMR experiments were acquired using standard Bruker library pulse sequences.

### Data availability

The data that support the findings of this study are available from the corresponding author upon request.

## Additional information

**How to cite this article**: Zavras, A. *et al*. Ligand-induced substrate steering and reshaping of [Ag_2_(H)]^+^ scaffold for selective CO_2_ extrusion from formic acid. *Nat. Commun.* 7:11746 doi: 10.1038/ncomms11746 (2016).

## Supplementary Material

Supplementary Information Supplementary Figures 1-19, Supplementary Tables 1-4, Supplementary Methods and Supplementary References
