# Supplementary material for: Ligand-induced substrate steering and reshaping of [Ag2(H)]+ scaffold for selective CO2 extrusion from formic acid
Source: Nat Commun. 2016 Jun 6;7:11746. doi: 10.1038/ncomms11746 (PMC4897753; doi:10.1038/ncomms11746)
Supplement: Supplementary Information — Supplementary Figures 1-19, Supplementary Tables 1-4, Supplementary Methods and Supplementary References [file ncomms11746-s1.pdf]

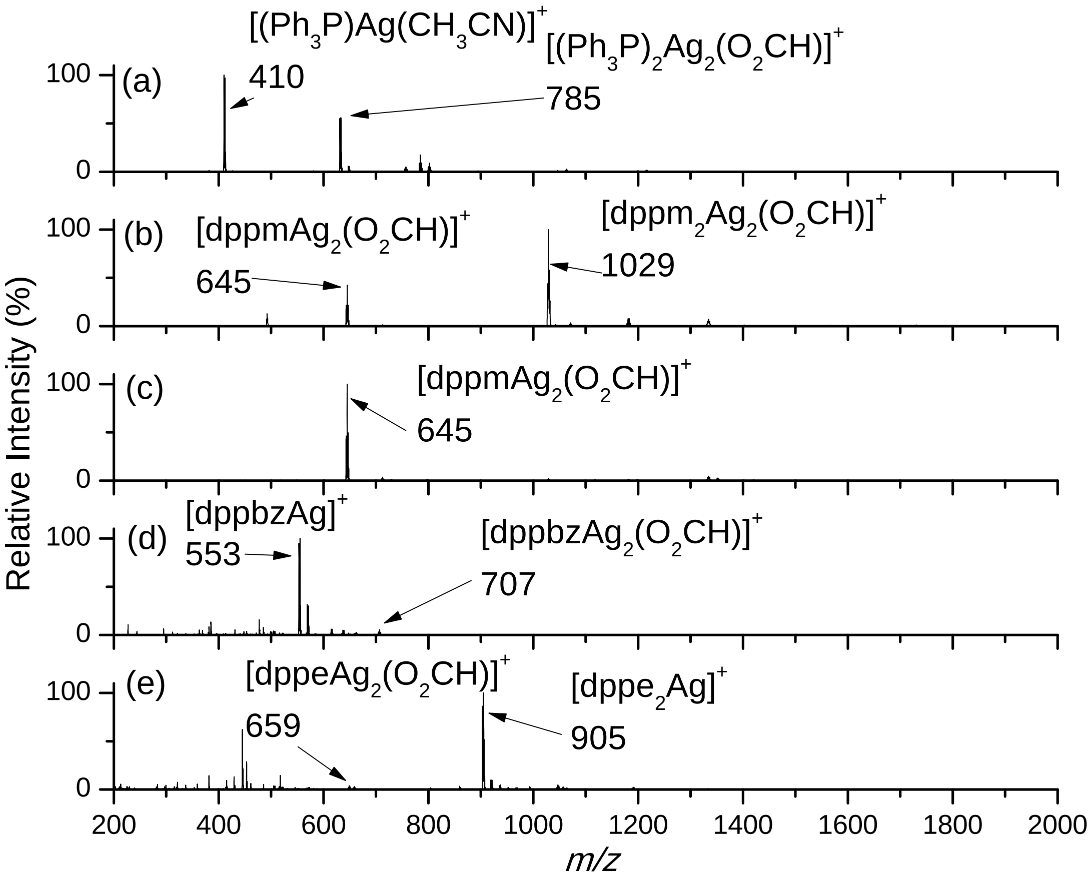

Supplementary Figure 1 | LTQ ESI-MS of ligated silver formate clusters formed in a 10 mL solution. (a) methanol:dichloromethane (1:1) of 20 mmol $AgNO_3$ and 10 mmol PPh_3 5 minutes after 10 mmol NaO_2CH was added; (b) acetonitrile:water (95:5) of 20 mmol $AgBF_4$, 10 mmol dppm and 10 mmol NaO_2CH; (c) methanol:formic acid (1:1) of 20 mmol $AgNO_3$, 10 mmol dppm and 10 mmol NaO_2CH; (d) dichloromethane:acetonitrile:formic acid (45:45:10) 20 mmol $AgNO_3$, 10 mmol dppm and 10 mmol NaO_2CH; (e) dichloromethane:acetonitrile:formic acid (45:45:10) of 20 mmol $AgNO_3$, 10 mmol dppm and 10 mmol NaO_2CH. Solutions containing silver clusters were diluted to 50 μM prior to ESI. The *m/z* represents the most abundant isotope peak in the cluster.

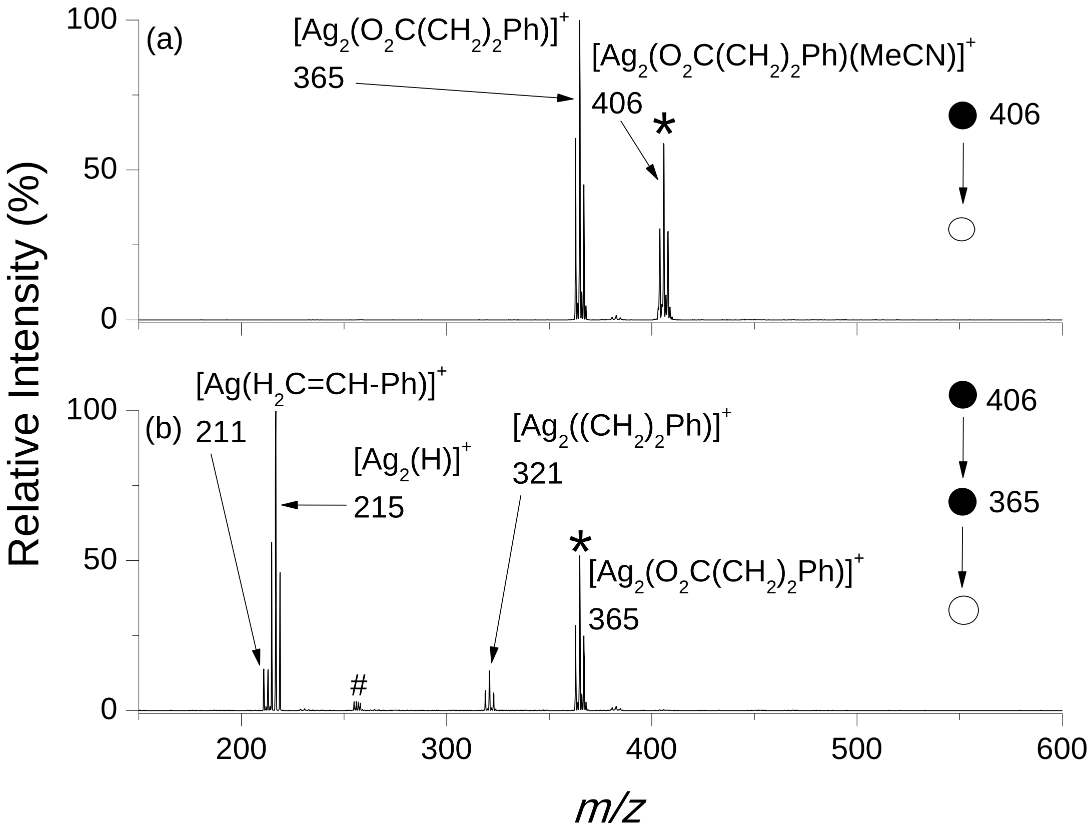

Supplementary Figure 2 | LTQ collision-induced dissociation of dinuclear silver clusters to generate $[Ag_2(H)]^+$ *m/z* 215. (a) $[Ag_2(O_2C(CH_2)_2Ph)(MeCN)]^+$ *m/z* 406, and (b) $[Ag_2(O_2C(CH_2)_2Ph)]^+$ *m/z* 365. The most intense peak in the cluster is represented by the *m/z* value. * Refers to the mass selected precursor cluster. # Refers to reaction with background solvent.

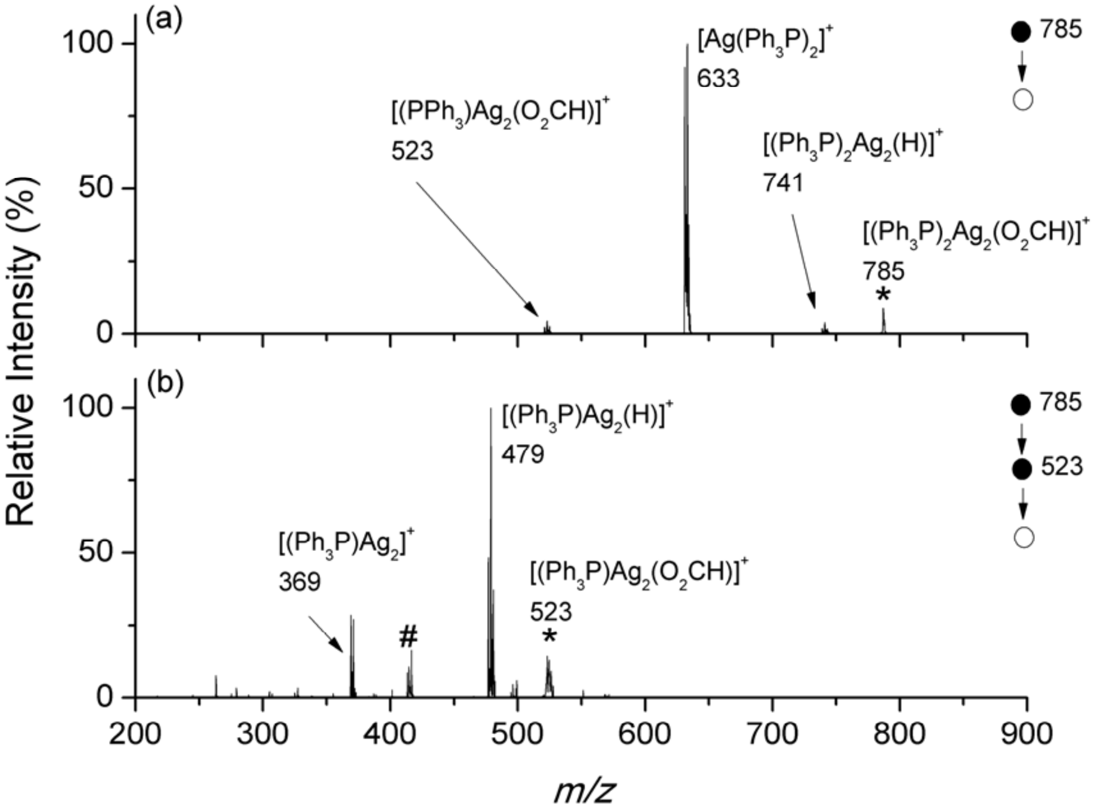

Supplementary Figure 3│LTQ collision-induced dissociation to generate $[(Ph_3P)Ag_2(H)]^+$ *m/z* 479. (a) $[(Ph_3P)_2Ag_2(O_2CH)]^+$ *m/z* 785. (b) $[(Ph_3P)Ag_2(O_2CH)]^+$ *m/z* 523. The most intense peak in the cluster is represented by the *m/z* value. * Refers to the mass selected precursor cluster.

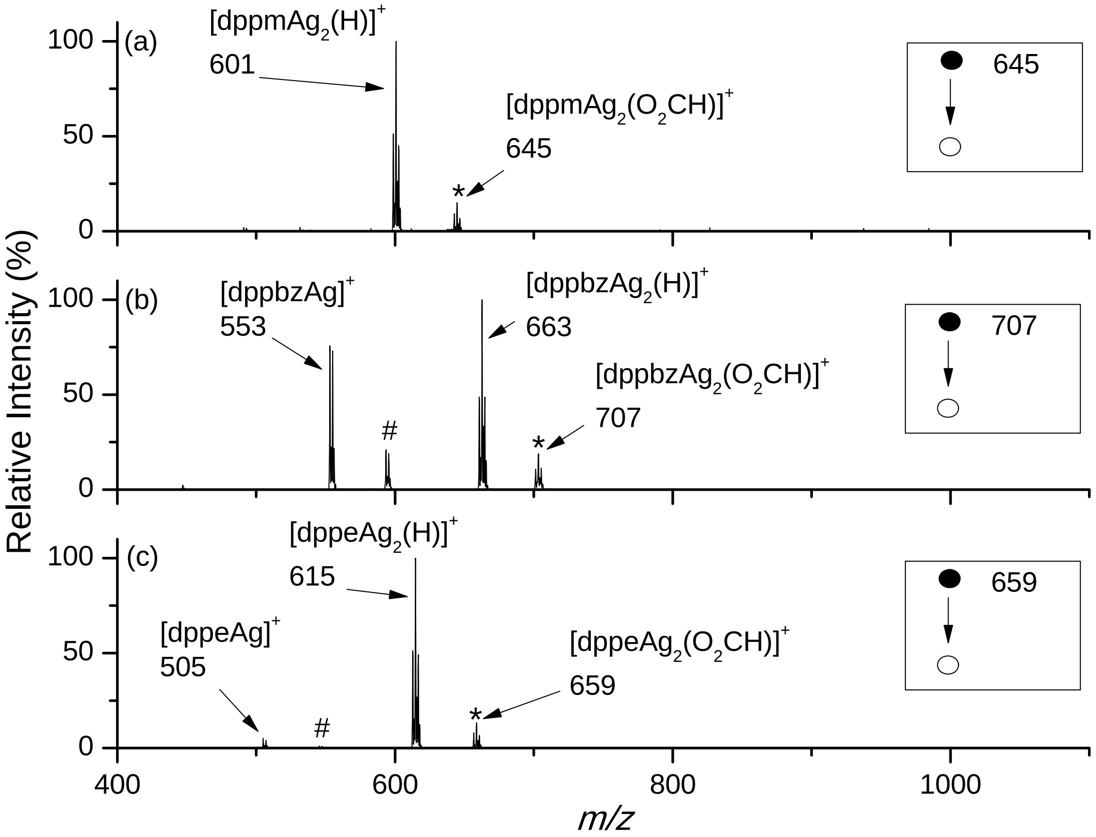

Supplementary Figure 4 | Collision-induced dissociation of [$LAg_2(O_2CH)]^+$. (a) L = bis(diphenylphosphino)methane (dppm); (b) 1,2-bis(diphenylphosphino)benzene (dppbz) and (b) bis(diphenylphosphino)ethane (dppe). The most intense peak in the ion is represented by the *m/z* value. * Represents the mass selected precursor ion. # Represents reaction of $[LAg]^+$ (L = dppbz and dppe) with background acetonitrile in the ion trap from the ESI solvent.

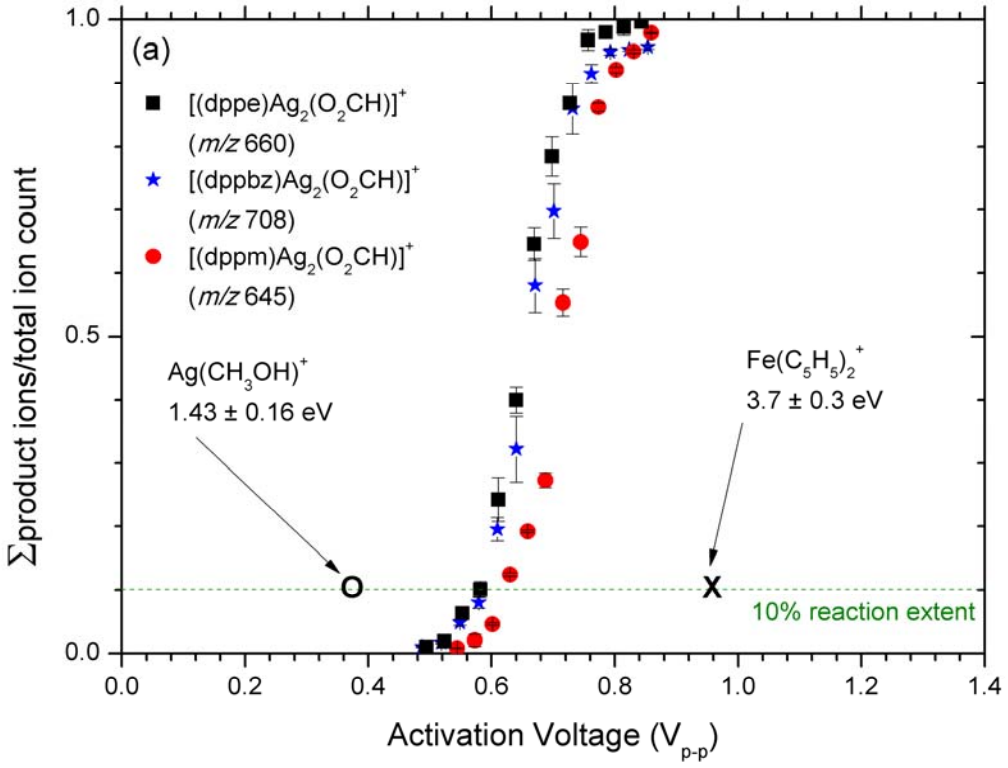

Supplementary Figure 5 | LCQ energy-resolved CID data for the decarboxylation of $[LAg_2(O_2CH)]^+$. (a) Plot of reaction extent (Σproduct ions/total ion count) vs activation voltage ($V_{p\text{-}p}$) for the decarboxylation of $[LAg_2(O_2CH)]^+$. The solid red circle corresponds to L = dppm = bis(diphenylphosphino)methane; the solid blue star corresponds to L = dppbz = 1,2-bis(diphenylphosphino)benzene, and; the solid black square corresponds to L = dppe = 1,2-bis(diphenylphosphino)ethane. Each coordinate is the average of at least three separate experiments from which the standard deviation is shown as error bars. The threshold activation voltages correspond to the dashed olive line at 10% reaction extent. The thresholds for dissociation were also measured for two systems were the critical energies for dissociation are known from other gas-phase experiments: $Ag(CH_3OH)^+$ (1.43 ± 0.16 eV) and $Fe(C_5H_5)_2^+$ (3.7 ± 0.3 eV).

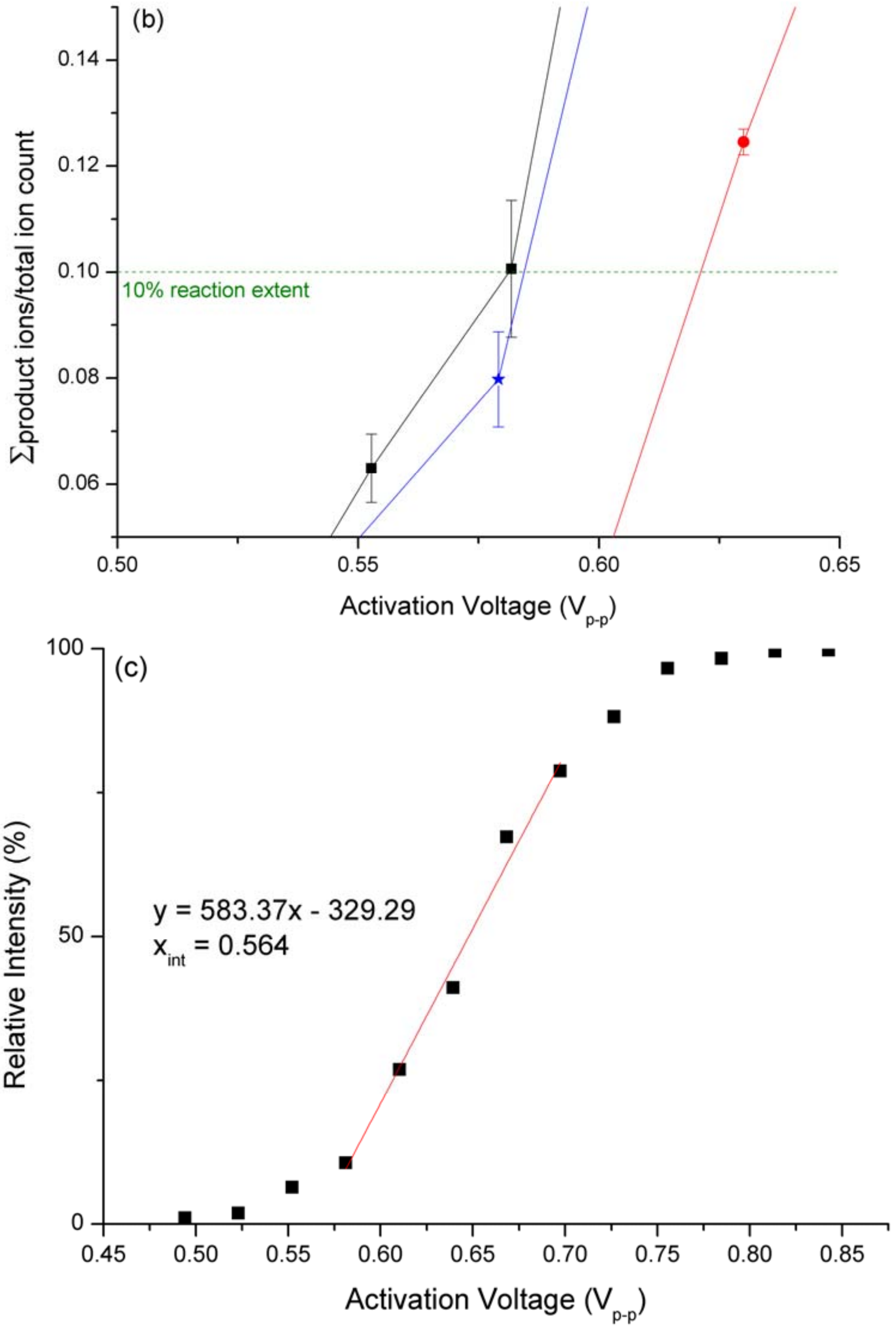

Supplementary Figure 5 | LCQ energy-resolved CID data for the decarboxylation of [LAg$_2$(O$_2$CH)]$^+$. (b) Expanded view of Figure S5a showing the corresponding activation voltage (V_{p-p}) for [dppeAg$_2$(O$_2$CH)]$^+$ (0.582) ≈ [dppbzAg$_2$(O$_2$CH)]$^+$ (0.585) < [dppmAg$_2$(O$_2$CH)]$^+$ (0.621) at 10% reaction extent. (c) Plot of relative abundance for the formation of [dppeAg$_2$(H)]$^+$ from the ERCID of **1f** as a function of activation voltage (V_{p-p}). Linear regression analyses using data points from 0.58 – 0.70 (V_{p-p}). R^2 = 0.98.

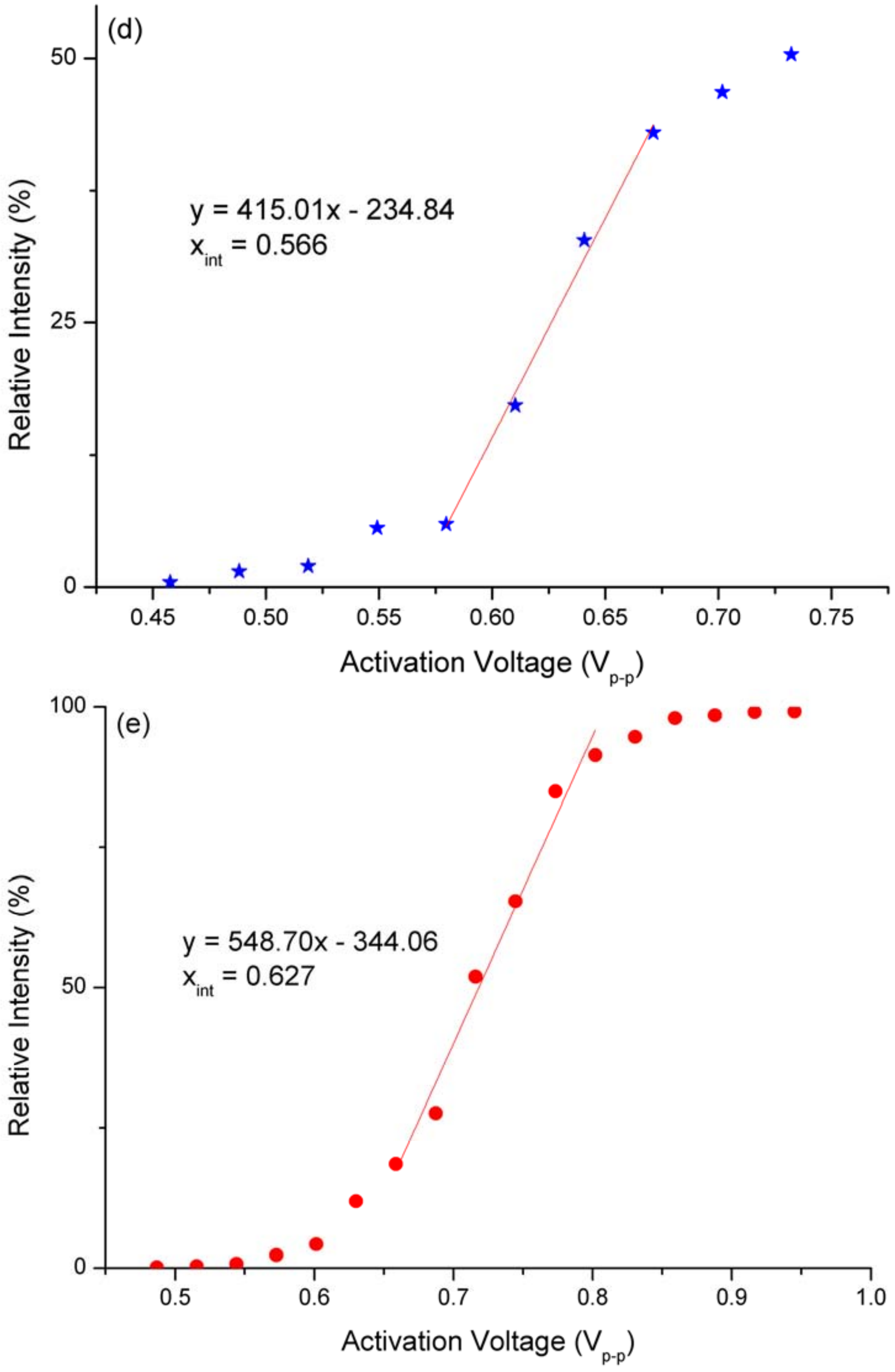

Supplementary Figure 5 | LCQ energy-resolved CID data for the decarboxylation of [LAg$_2$(O$_2$CH)]$^+$. (d) Plot of relative abundance for the formation of [dppbzAg$_2$(H)]$^+$ from the ERCID of **1e** as a function of activation voltage ($V_{p\text{-}p}$). Linear regression analyses using data points from 0.58 – 0.67 ($V_{p\text{-}p}$). R^2 = 0.99. (e) Plot of relative abundance for the formation of [dppmAg$_2$(H)]$^+$ from the ERCID of **1d** as a function of activation voltage ($V_{p\text{-}p}$). Linear regression analyses using data points from 0.66 – 0.80 ($V_{p\text{-}p}$). R^2 = 0.98.

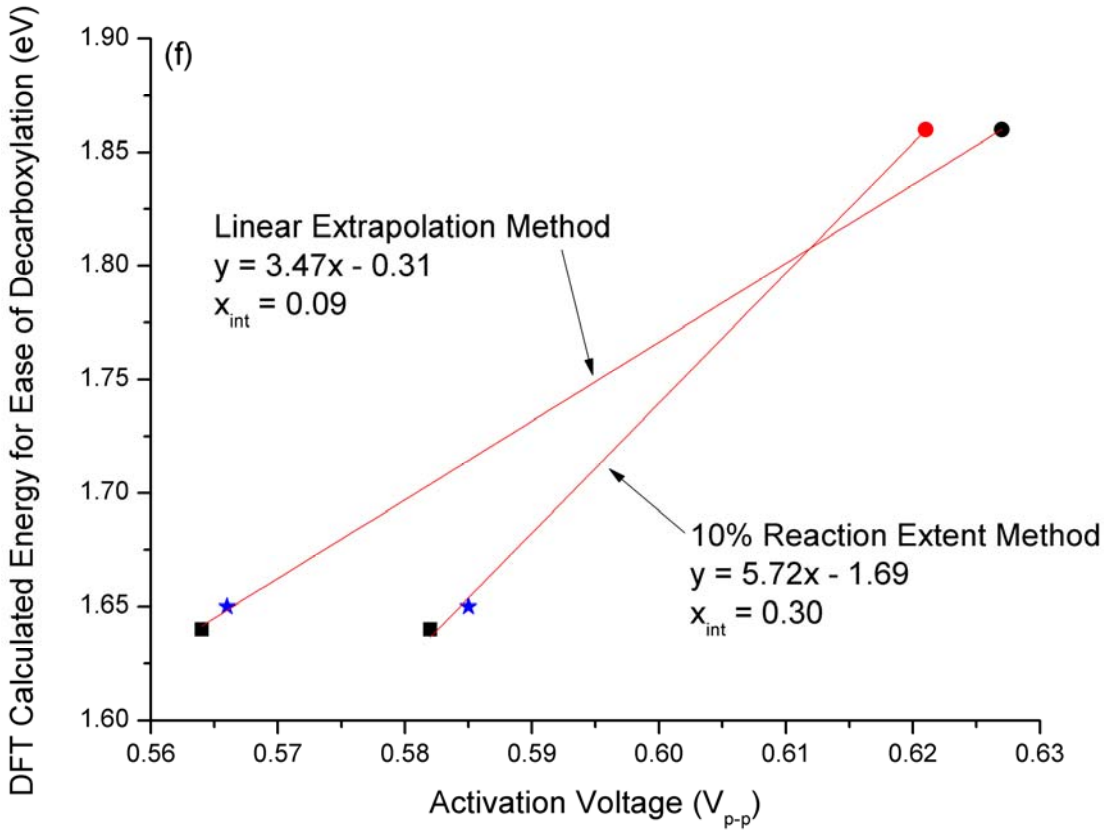

Supplementary Figure 5│LCQ energy-resolved CID data for the decarboxylation of $[LAg_2(O_2CH)]^+$. ((f) Comparison of the experimentally determined activation voltage (V_{p-p}) by the 10% reaction extent method and the linear regression analyses method (x-axis) vs. the DFT calculated energy (eV) for ease of decarboxylation (y-axis) for the decarboxylation transition states given in Supplementary table 3.

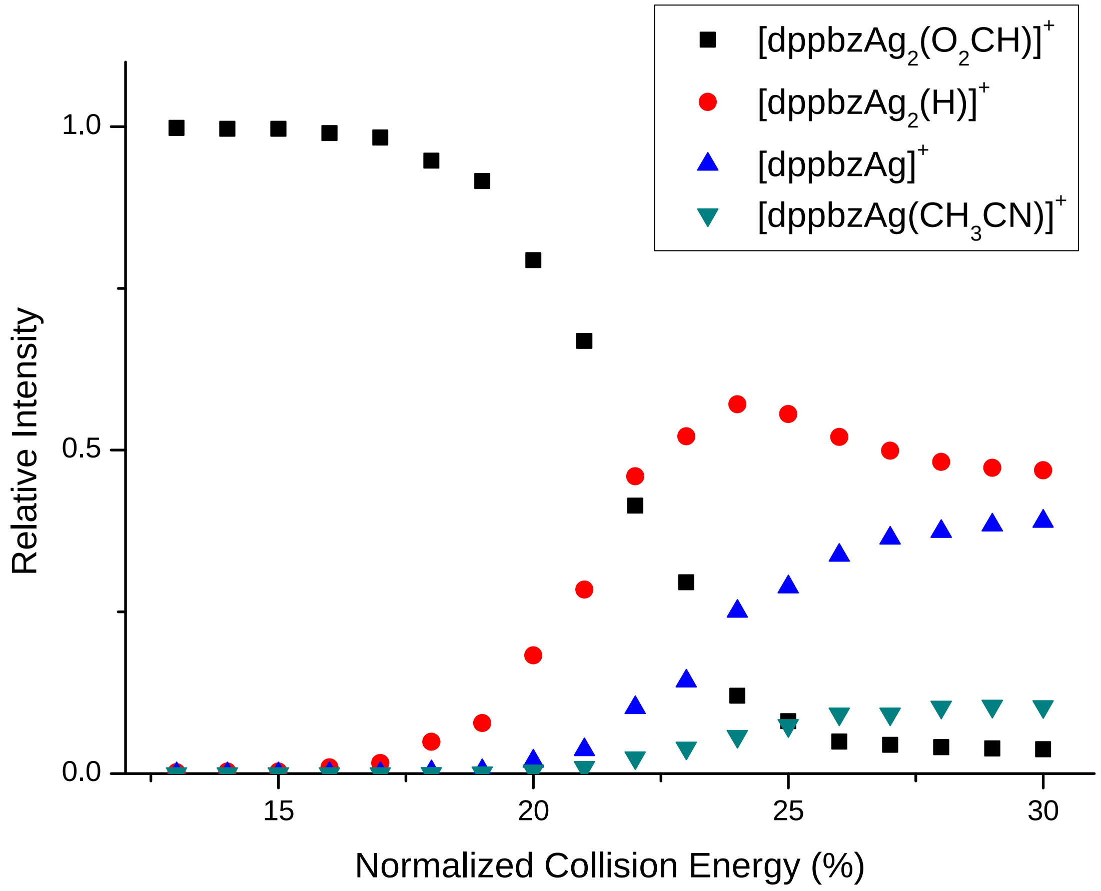

Supplementary Figure 6 | LCQ energy-resolved CID of [dppbzAg$_2$(O$_2$CH)]$^+$. Branching ratios as a function of CID energy.

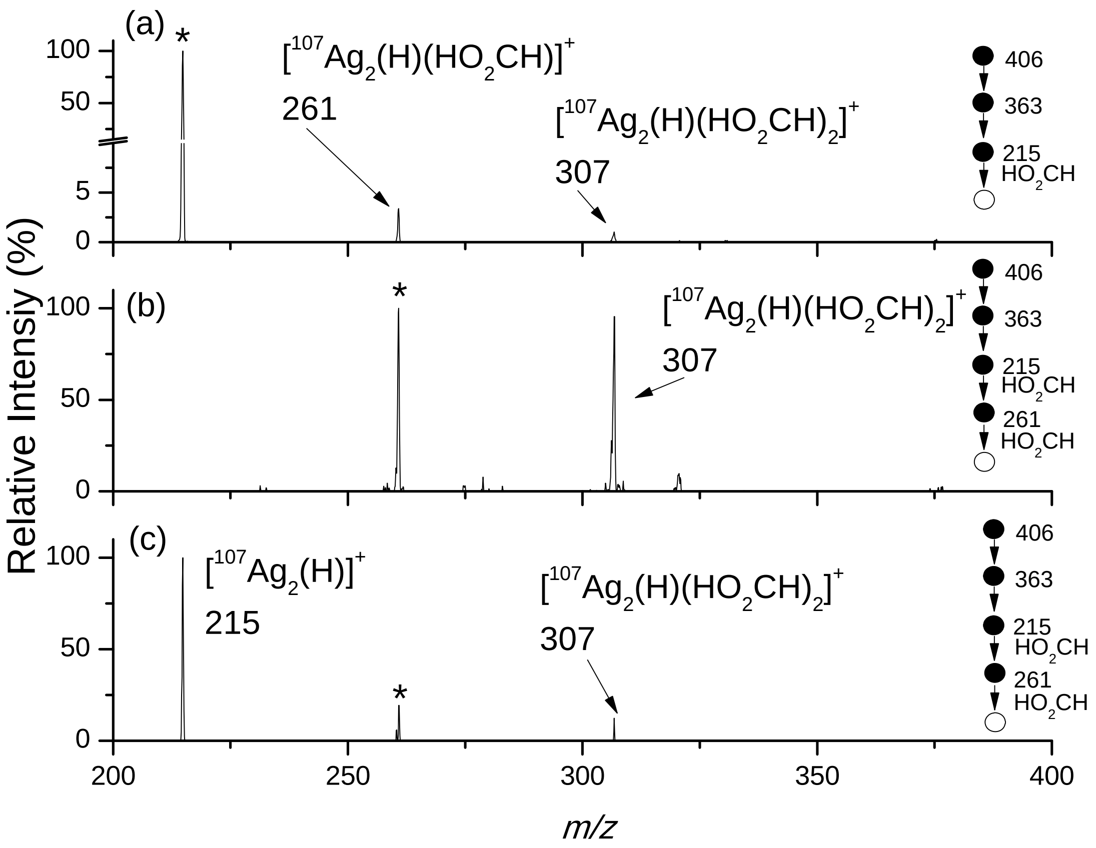

Supplementary Figure 7│LTQ-FT gas-phase reactions of silver hydrides, $[Ag_2(H)]^+$, with formic acid. (a) ion-molecule reaction of $[^{107}Ag_2(H)]^+$ *m/z* 215 with formic acid for 1000 ms; (b) ion-molecule reaction of $[^{107}Ag_2(H)(HO_2CH)]^+$ *m/z* 261 with formic acid for 5000 ms; (c) CID of $[^{107}Ag_2(H)(HO_2CH)]^+$ *m/z* 215. The most intense peak in the cluster is represented by the *m/z* value. * Refers to the mass selected precursor cluster. # Refers to noise peaks.

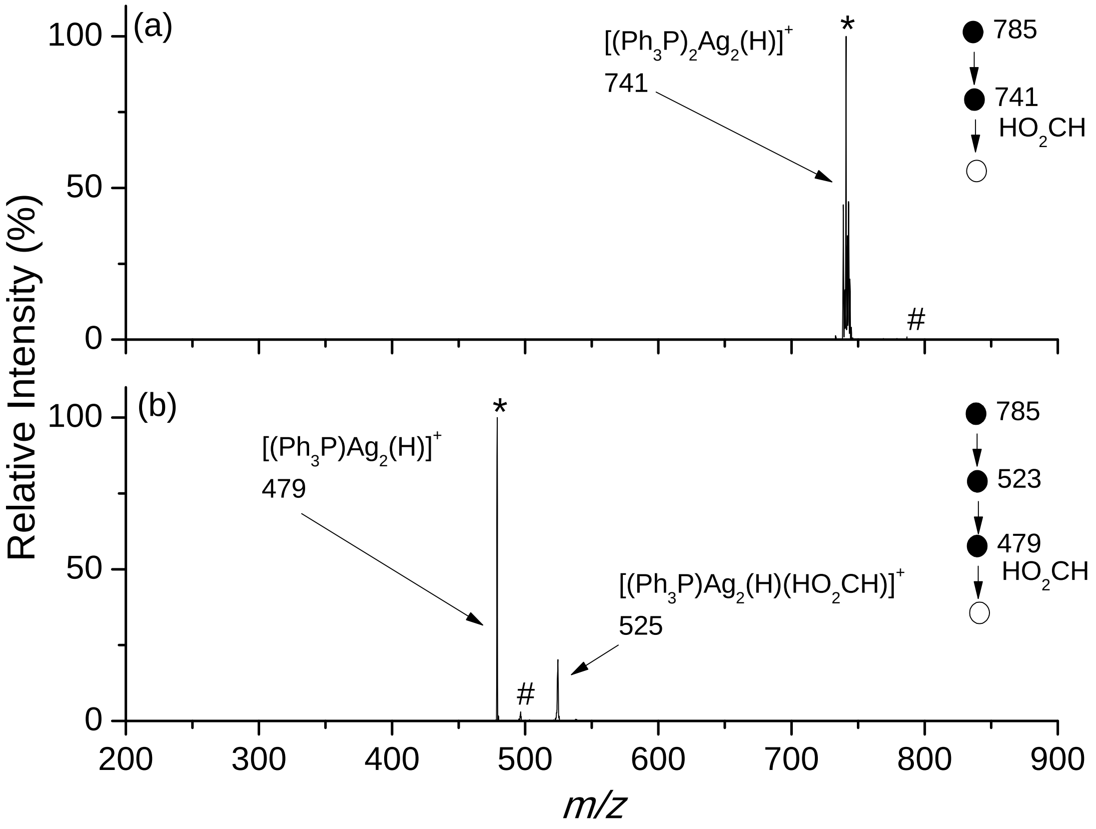

Supplementary Figure 8│LTQ-FT ion-molecule reaction of dinuclear silver hydrides coordinated to triphenylphosphine (PPh_3) with formic acid. (a) $[(Ph_3P)Ag_2(H)]^+$ *m/z* 741 for 10000 ms. (b) LTQ ion-molecule reaction of $[(Ph_3P)Ag_2(H)]^+$ *m/z* 479 for 100 ms. The most intense peak in the cluster is represented by the *m/z* value. * Refers to the mass selected precursor cluster. # Refers to noise peaks.

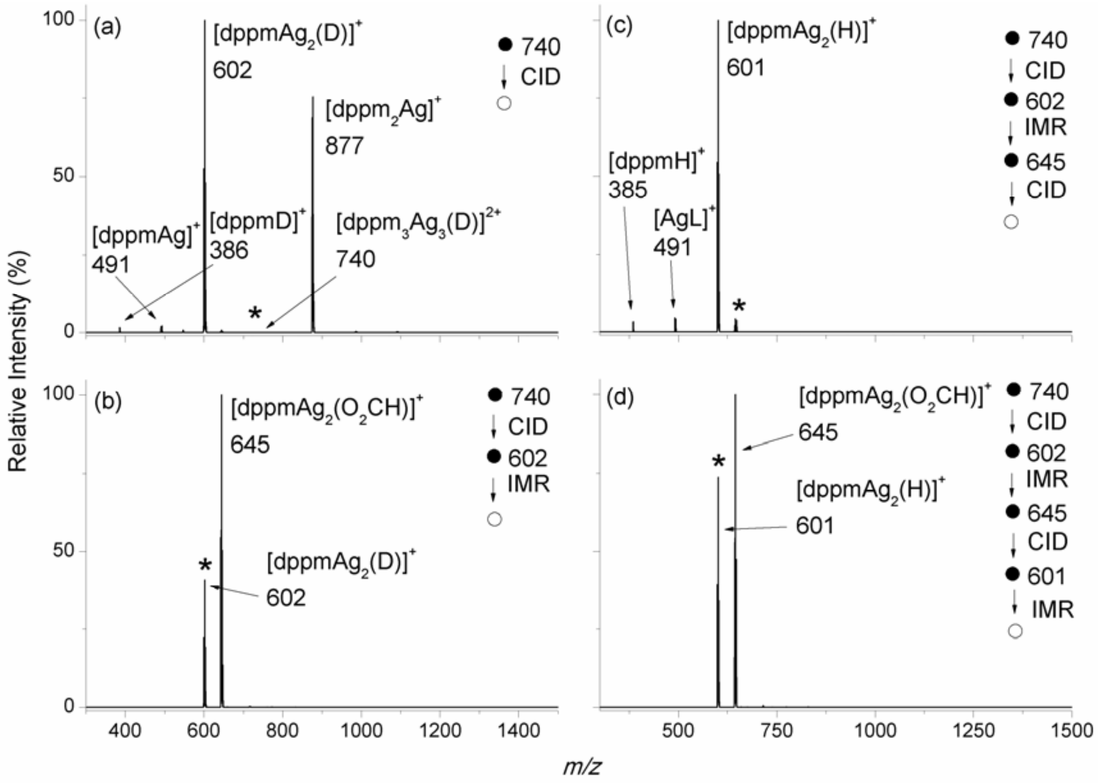

Supplementary Figure 9│Synthesis of deuterium labelled [dppmAg$_2$(D)]$^+$ and reaction with formic acid. (a) LTQ collision-induced dissociation of [dppm$_3$Ag$_3$(D)]$^{2+}$ *m/z* 740, prepared from [Ag$_3$(μ$_3$-D)dppm$_3$](BF$_4$)$_2$. (b) LTQ ion-molecule reaction of [dppmAg$_2$(D)]$^+$ *m/z* 602 with HO$_2$CH for 1000 ms. (c) LTQ collision-induced dissociation of [dppmAg$_2$(O$_2$CH)]$^+$ *m/z* 645. (c) LTQ ion-molecule reaction of [dppmAg$_2$(H)]$^+$ *m/z* 601 with HO$_2$CH for 1000 ms. The most intense peak in the cluster is represented by the *m/z* value. * Refers to the mass selected precursor cluster.

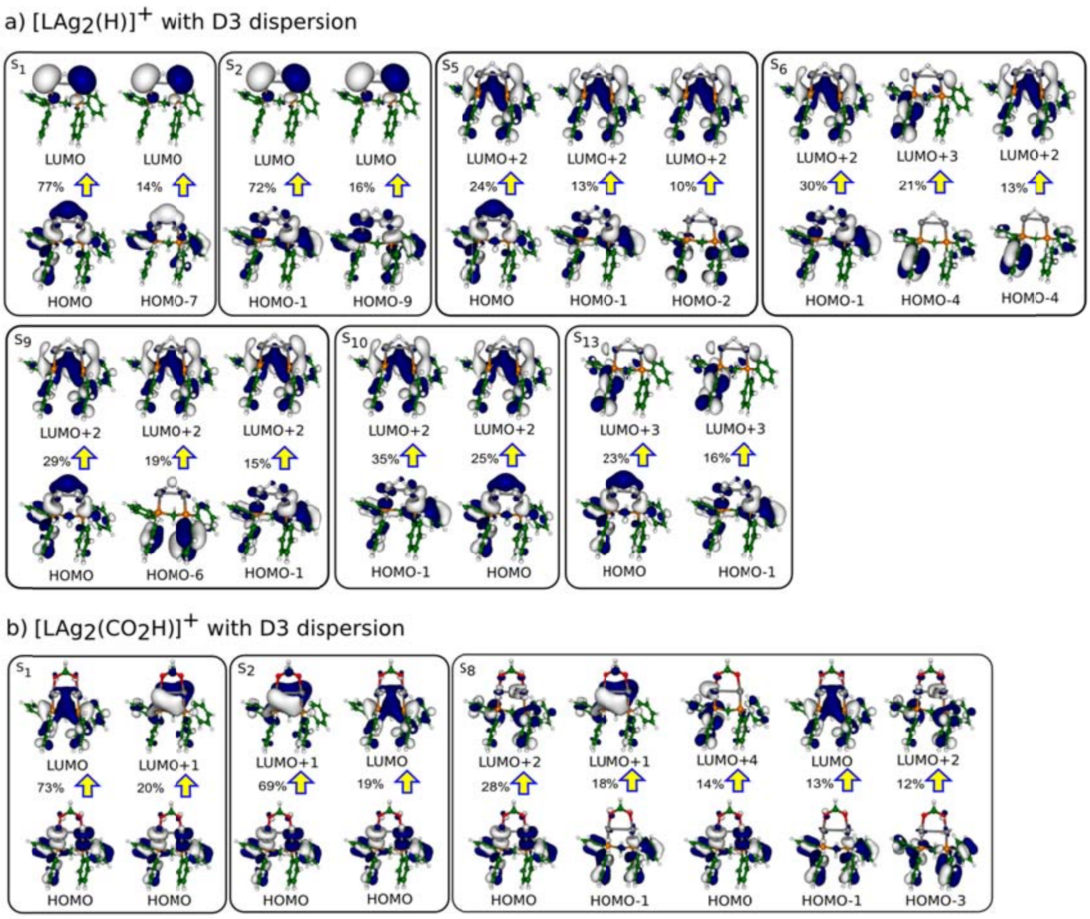

Supplementary Figure 10 | The analysis of leading excitations contributing to the most intense transitions of UV absorption spectra shown in Figures 4c and 4d. (a) $[dppmAg_2(H)]^+$ and (b) $[dppmAg_2(O_2CH)]^+$ respectively; cut-off for MOs is 0.04, minus and plus are labeled by blue and grey colors, respectively.

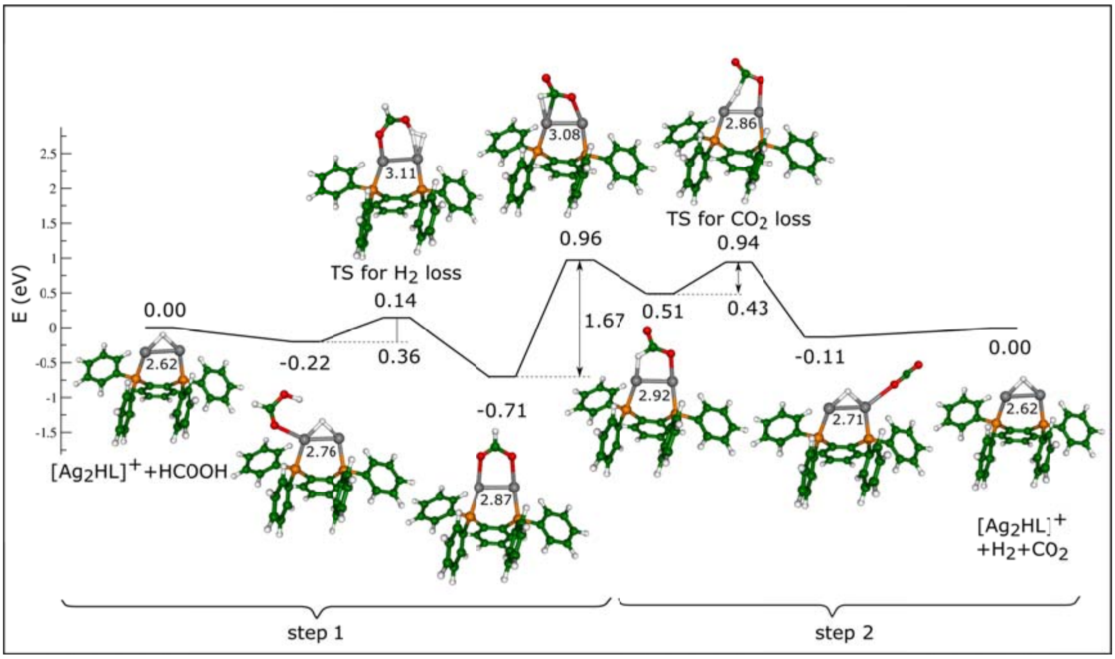

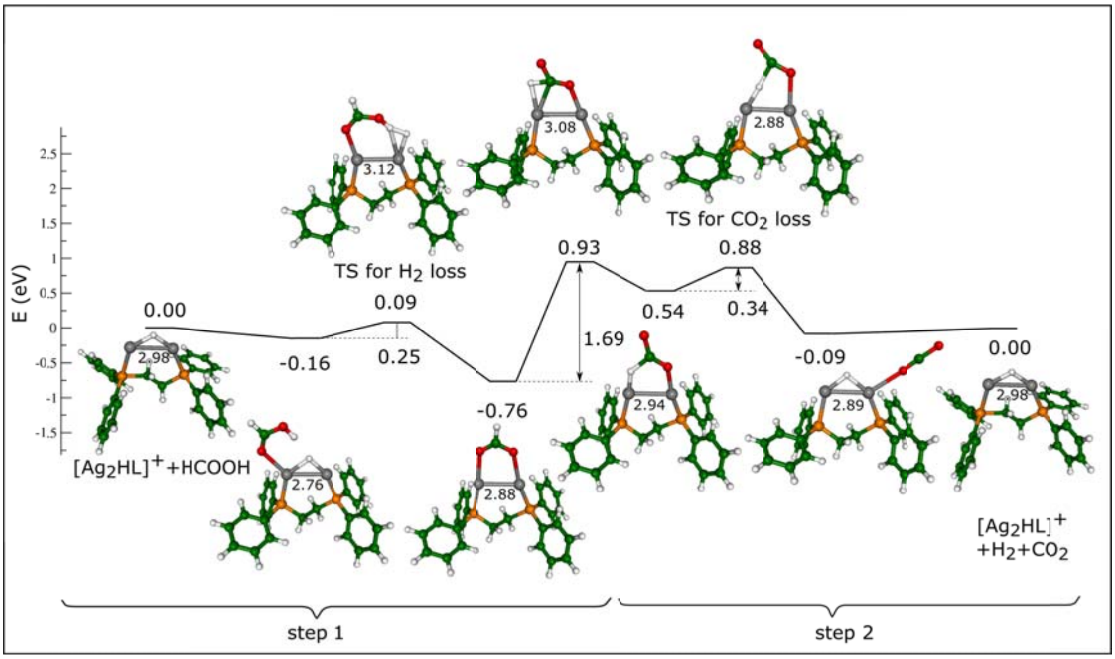

Supplementary Figure 11 | DFT-calculated energy profile for the two reaction steps in the catalytic cycle for: (a) **1e** and (b) **1f**.

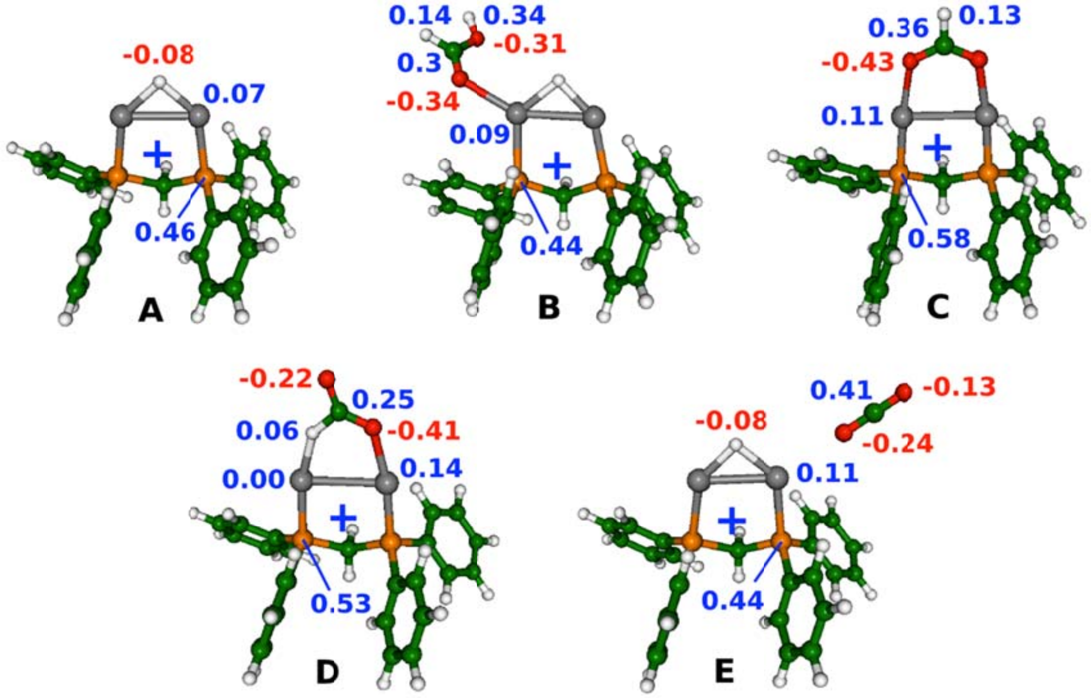

Supplementary Figure 12 | Calculated charge distribution. (positive charge blue, negative charge red), blue lines close to atoms label positive charge and the corresponding region in which the charge is delocalized is denoted by (+). Bond distances are given in Å (black).

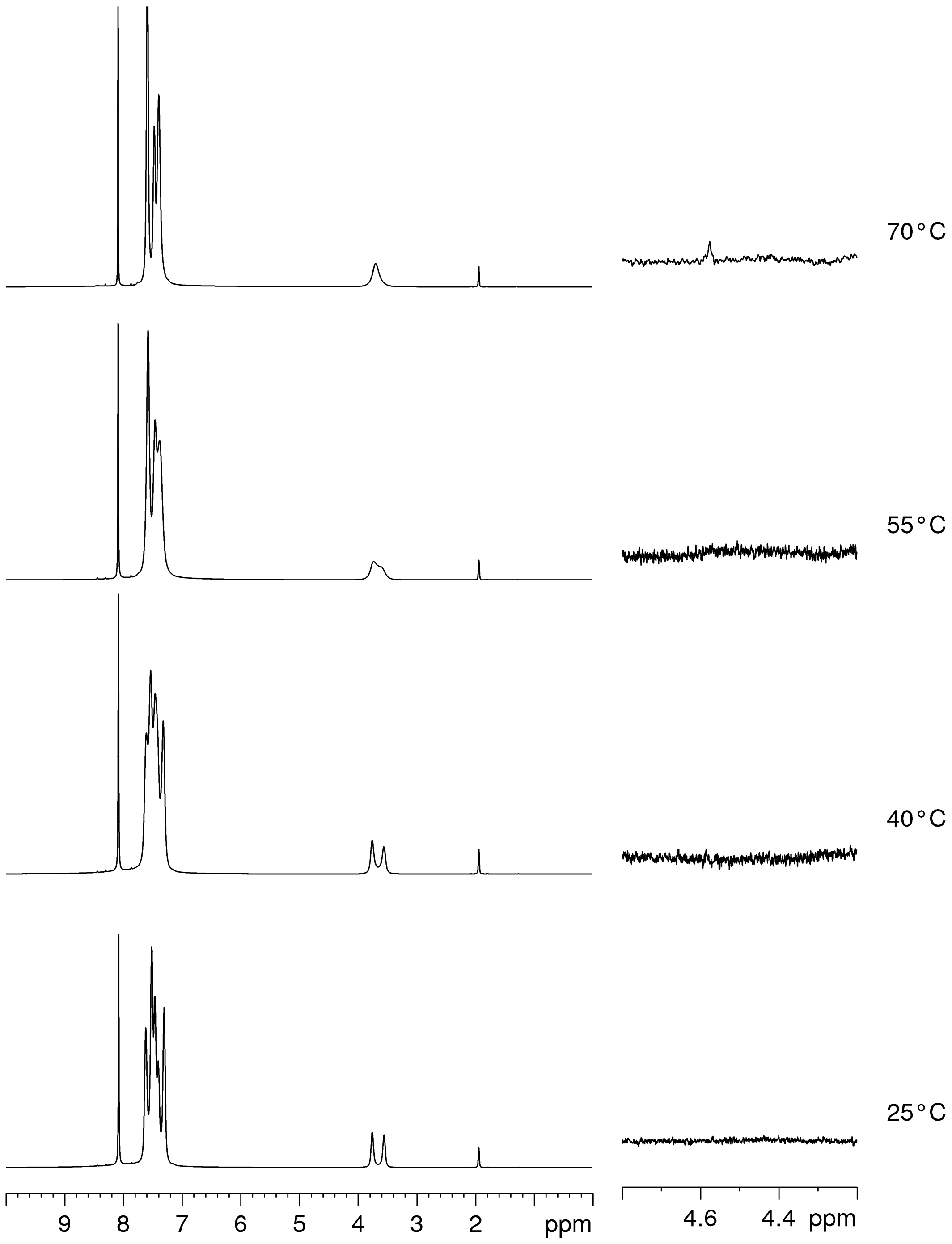

Supplementary Figure 13 | Variable temperature (+25 – +70°C) ^{1}H NMR of Ag, dppm, formic acid, sodium formate in CD_3CN. Note the evolution of H_2 gas at 4.65 ppm.

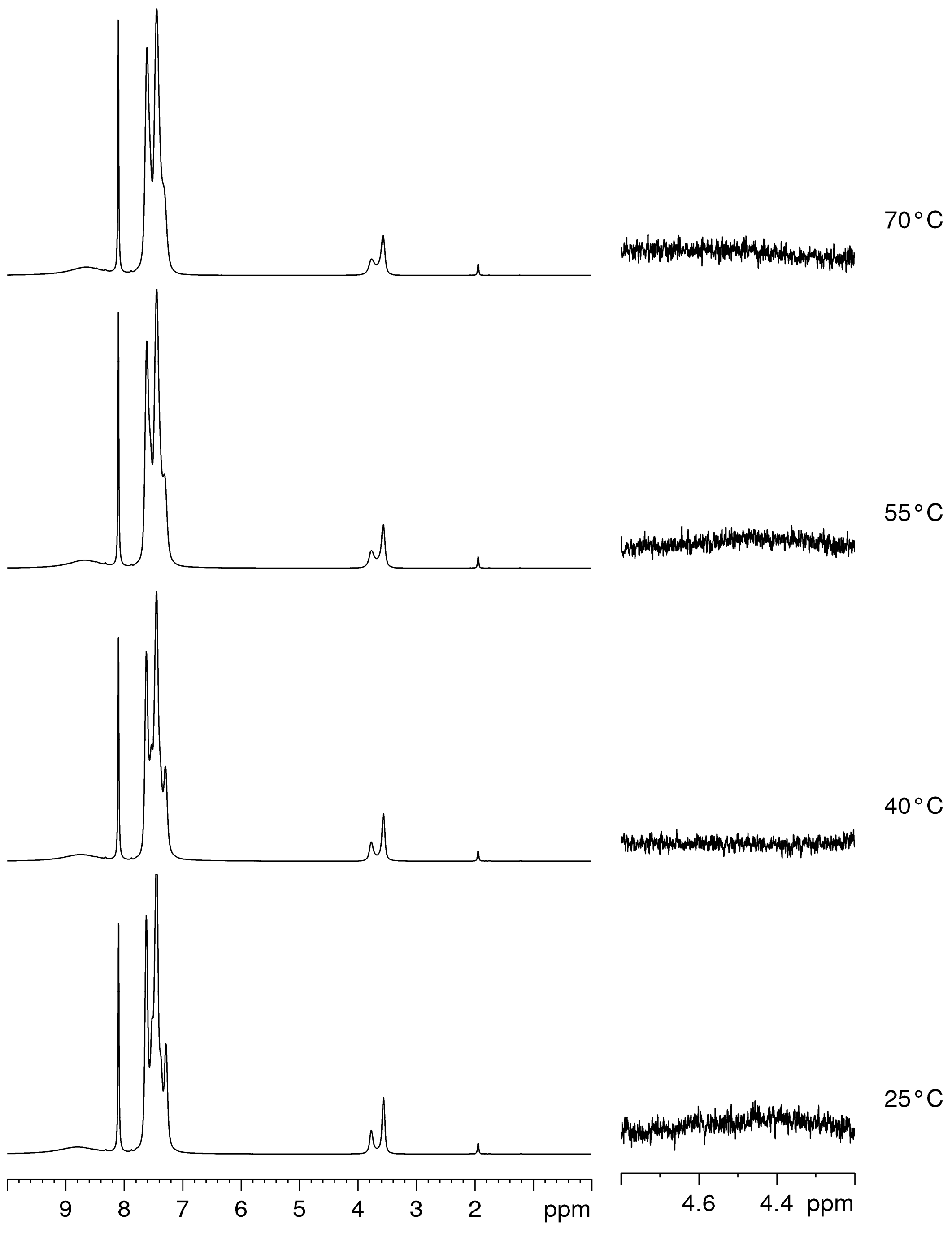

Supplementary Figure 14 | Variable temperature (+25 – +70°C) ^{1}H NMR of Ag, dppm, formic acid in CD_3CN. Note that no H_2 gas is evolved at 4.65 ppm.

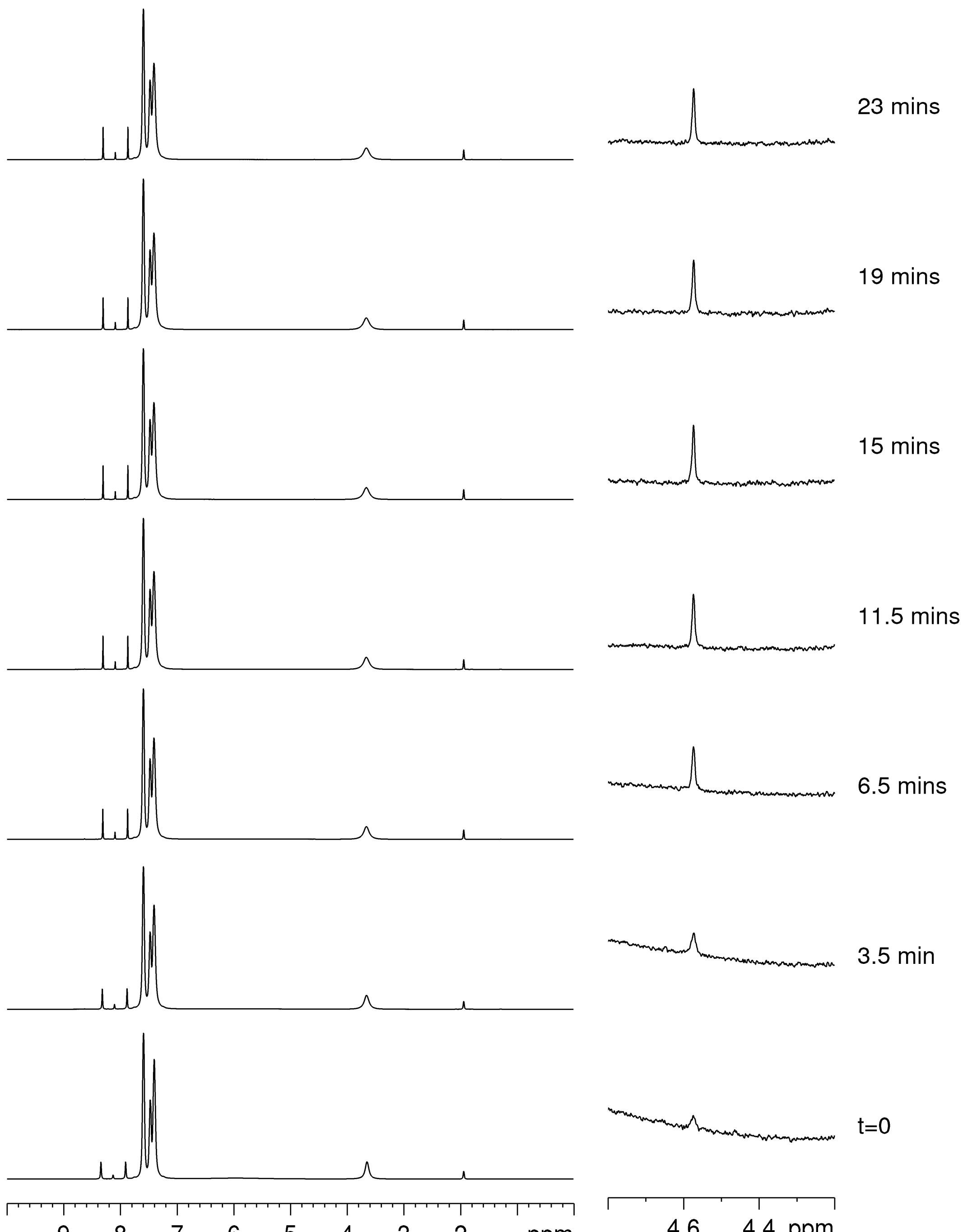

Supplementary Figure 15 | ^{1}H NMR at 70°C of Ag, dppm, ^{13}C-formic acid and sodium formate in CD_3CN. Note the evolution of H_2 gas at 4.6 ppm.

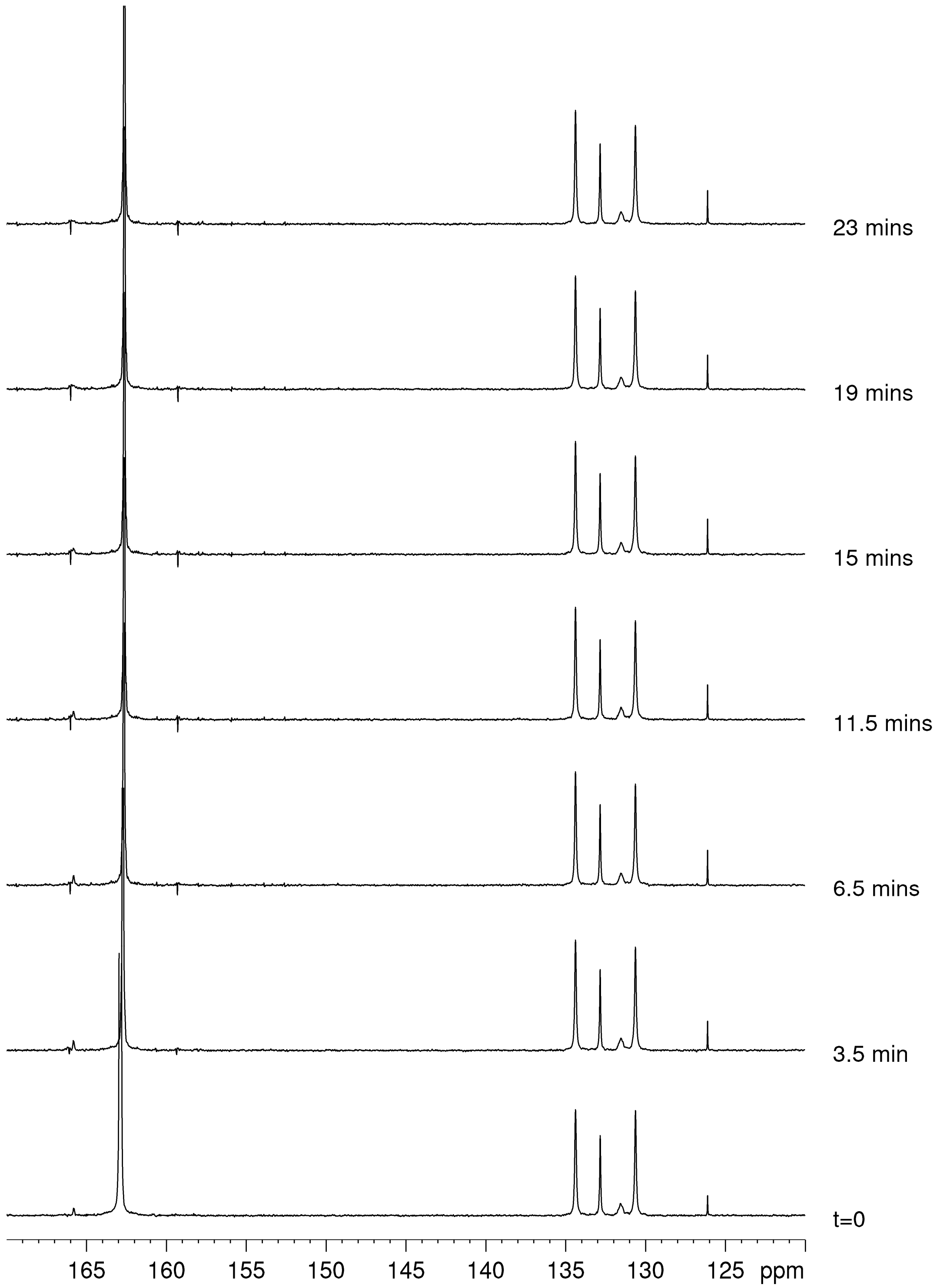

Supplementary Figure 16 | ^{13}C NMR at 70°C of Ag, dppm, ^{13}C-formic acid and sodium formate in CD_3CN. Note the evolution of $^{13}CO_2$ gas at 126 ppm.

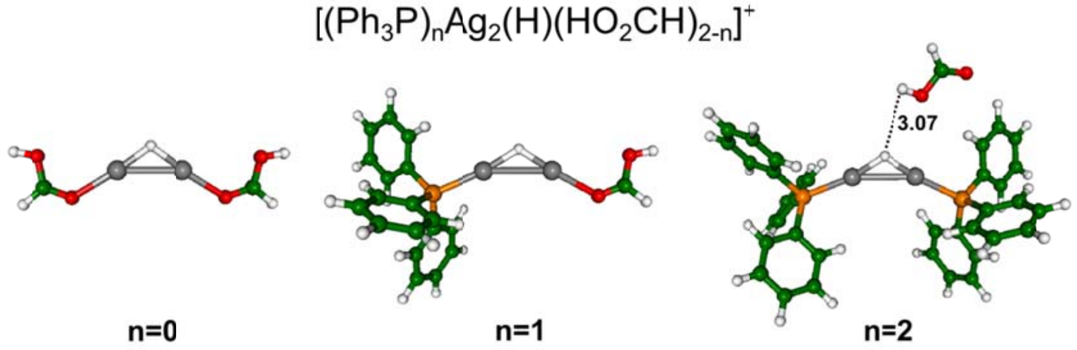

Supplementary Figure 17 | The most stable DFT calculated structures for the interaction of formic acid with $[(Ph_3P)_nAg_2(H)(HO_2CH)_{2-n}]^+$. For n = 0, both formic acid molecules coordinate to a separate Ag site. For n = 1, the formic acid coordinates at the vacant coordination site. For n = 2, there is not vacant coordination site and so the formic acid is weakly bound in an ion-molecule complex. Note the formic acid molecules are in trans conformation.

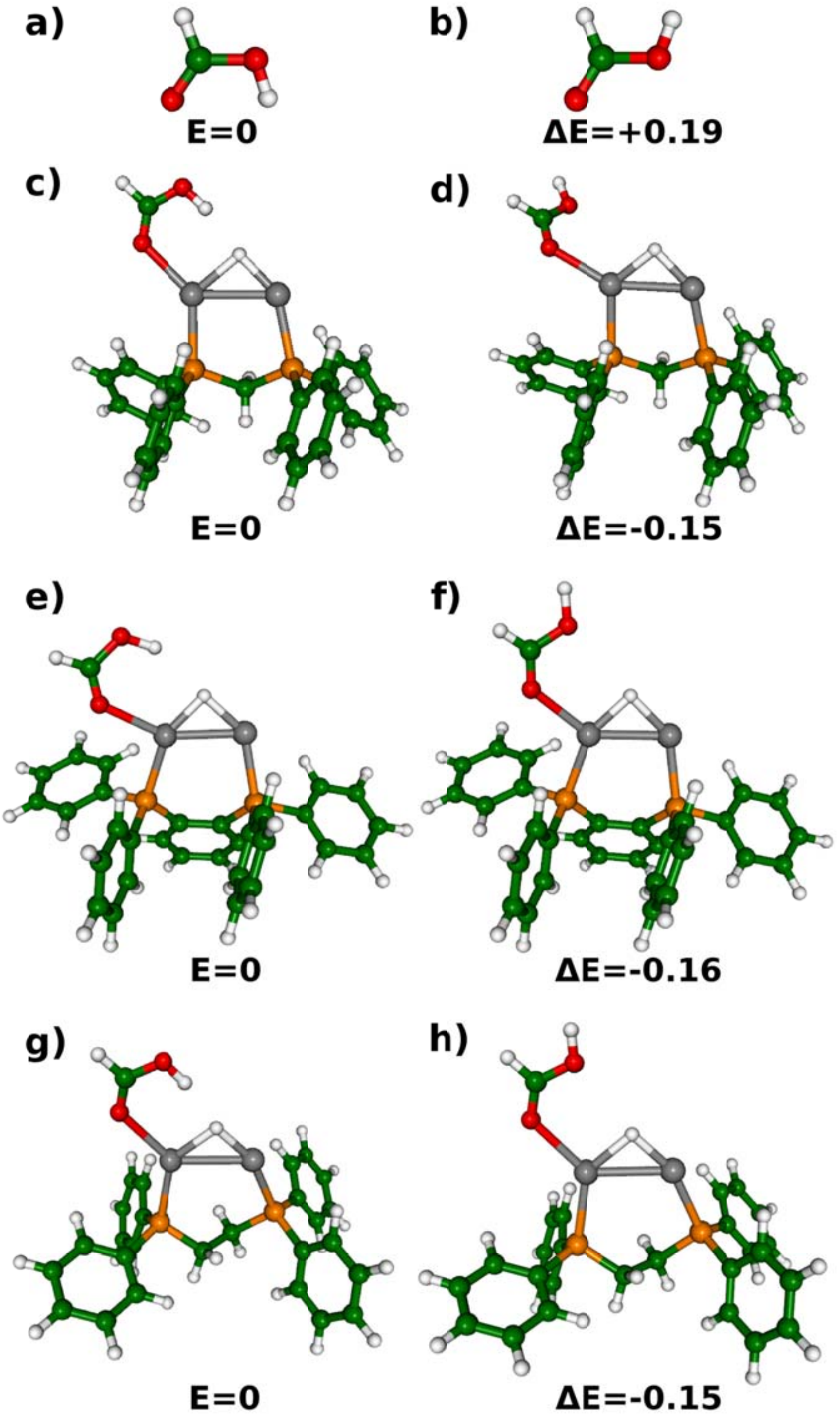

Supplementary Figure 18 | DFT calculated structures and relative energies of cis versus trans conformers of formic acid and their adducts with $[LAg_2(H)]^+$: (a) cis formic acid; (b) trans formic acid; (c) cis formic acid adduct of **1d** (dppm); (d) trans formic acid adduct of **1d** (dppm); (e) cis formic acid adduct of **1e** (dppbz); (f) trans formic acid adduct of **1e** (dppbz); (g) cis formic acid adduct of **1f** (dppe); (h) trans formic acid adduct of **1f** (dppe).

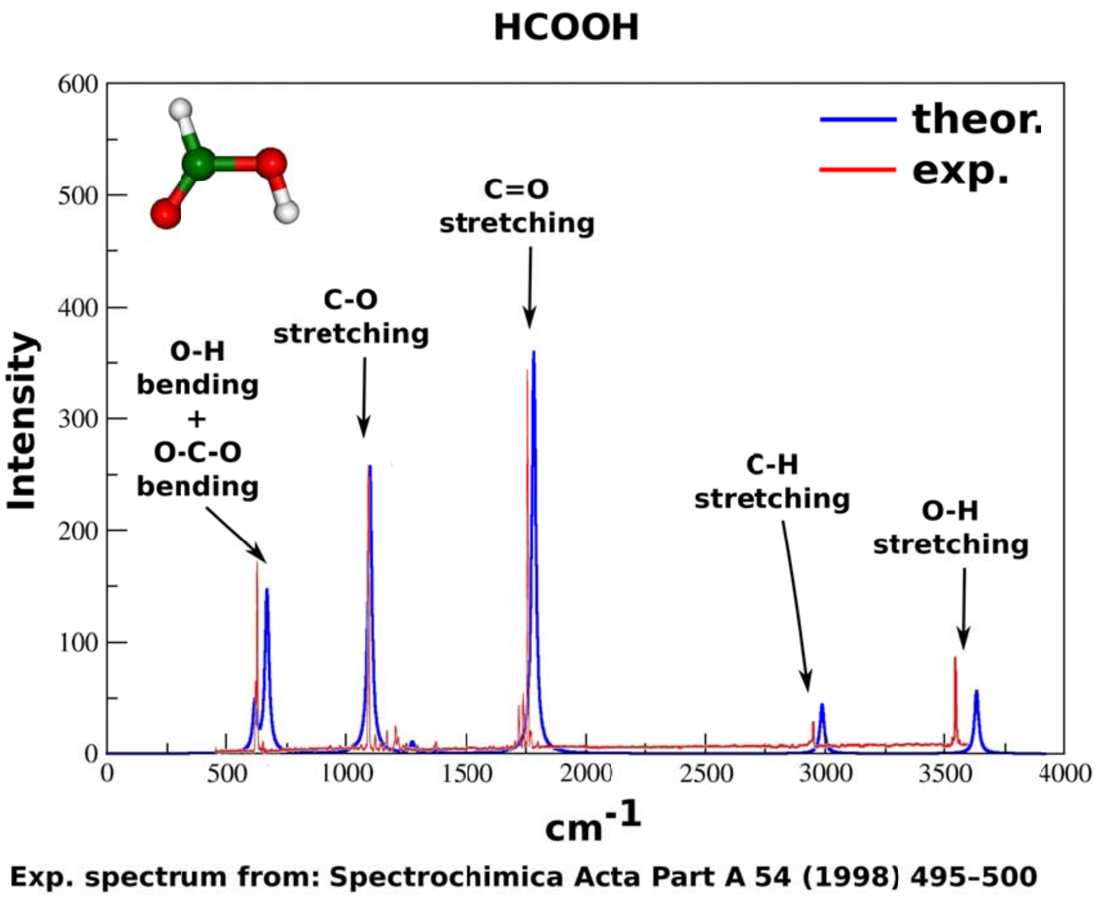

Supplementary Figure 19 | Experimental (matrix isolation) vs DFT calculated gas-phase IR scaled by 0.98 of monomeric formic acid (in cis conformation [5]).

Supplementary Table 1 | Kinetic data associated with the ion-molecule dinuclear silver hydrides with formic acid. 1a, $[Ag_2(H)]^+$; $[LAg_2(H)]^+$, where L = Ph_3P, **1b**; 2 x PPh_3, **1c**; dppm, **1d**; dppb, **1e** and; dppe, **1f** with formic acid.

**Reactant**	**Product**	**k_{expt}[a,b]**	**Reaction Efficiency[c]**
**1a** $[Ag_2(H)]^+$	$[Ag_2(H)(HO_2CH)]^+$	$1.25 \times 10^{-11} \pm 1.53 \times 10^{-13}$	1.12 ± 0.014
**1b** $[(Ph_3P)Ag_2(H)]^+$	$[(Ph_3P)Ag_2(H)(HO_2CH)]^+$	$8.45 \times 10^{-10} \pm 8 \times 10^{-12}$	79.52 ± 0.75
**1c** $[(Ph_3P)_2Ag_2(H)]^+$	N.R.[d]	N.A.	N.A.
**1d** $[dppmAg_2(H)]^{+}$[f]	$[dppmAg_2(O_2CH)]^+$	$1.53 \times 10^{-11} \pm 2.83 \times 10^{-13}$	1.45 ± 0.027
**1e** $[dppbzAg_2(H)]^{+}$[f]	$[dppbzAg_2(O_2CH)]^+$	$1.25 \times 10^{-11} \pm 6.18 \times 10^{-13}$	1.19 ± 0.059
**1f** $[dppeAg_2(H)]^{+}$[f]	$[dppeAg_2(O_2CH)]^+$	$2.27 \times 10^{-14} \pm 6.74 \times 10^{-16}$	$2.15 \times 10^{-3} \pm 6.4 \times 10^{-5}$

[a] In units of $cm^3.molecules^{-1}.s^{-1}$.

[b] Rates for the reaction with formic acid with $[LAg_2(H)]^+$ to regenerate $[LAg_2(O_2CH)]^+$ as the product. Rates were determined by monitoring the ion evolution with time.

[c] Reaction efficiency = $(k_{expt}/k_{ADO}) \times 100$. The k_{ADO} is the theoretical ion-molecule collision rate constant obtained from the average-dipole orientation (ADO) theory[6], which was calculated using the Colrate program[7].

[d] No reaction. The data shown for each reaction is the average of three separate experiments using different concentrations of neutral reagent.

Supplementary Table 2 | Assignment of experimentally observed vibrational bands of the [(dppm)$Ag_2(O_2CH)]^+$ (*m/z* 645) and [(dppm)$Ag_2(H)]^+$ (*m/z* 601) ions. (a) the position of the experimental bands are given in cm^{-1}. (b) Harmonic frequencies were scaled by 0.98, and predicted intensities (in parentheses) are given in $km.mol^{-1}$. Highlighted in green are the modes associated with the auxiliary ligands, and highlighted in red are the most structurally diagnostic band.

Mode Assignment	[(dppm)$Ag_2(O_2CH)]^+$ (*m/z* 645)		[(dppm)$Ag_2(H)]^+$ (*m/z* 601)	
	**Exp.**[a)]	**Theory**[b)]	**Exp.**[a)]	**Theory**[b)]
Ag as. st.			900	916 (245)
C_6H_5 in plane ring deformation	not observed	998 (9)<br>998 (10)<br>999 (5)<br>1000 (6)	998	998 (8)<br>998 (9)<br>999 (4)<br>1000 (6)
CH_2 twist; P-C_6H_5 st. (also C_6H_5 in plane CH bending and ring deformation)	1103	1086 (1)<br>1087 (14)<br>1087 (11)<br>1088 (16)<br>1090 (17)<br>1093 (33)<br>1094 (19)<br>1099 (19)	1097	1085 (6)<br>1086 (1)<br>1087 (2)<br>1088 (24)<br>1089 (30)<br>1091 (17)<br>1093 (26)<br>1099 (9)
AgH s. st.			1250	1236 (150)
formate s. CO st.	1360	1345 (121)		
CH_2 scissoring<br>C_6H_5 in plane CH bending	not observed	1393 (12)<br>1444 (11)<br>1445 (19)<br>1445 (9)<br>1446 (31)	1438	1395 (13)<br>1443 (13)<br>1444 (22)<br>1445 (9)<br>1445 (21)
C_6H_5 in plane CH bending and ring deformation	not observed	1490 (7)<br>1490 (5)<br>1492 (4)<br>1492 (7)	1478	1489 (7)<br>1489 (6)<br>1491 (3)<br>1491 (7)
formate as. CO st.	1547	1564 (504)		

Supplementary Table 3 | DFT (and DFT-D3) -calculated energies (eV) for steps 1 and 2 relative to the initial reactants.

		The energies (E) of two reaction steps in the catalytic cycle of $[LAg_2(H)]^+$		
		**1d** $[dppmAg_2(H)]^+$	**1e** $[dppbzAg_2(H)]^+$	**1f** $[dppeAg_2(H)]^+$
		**Relative E (eV)**	**Relative E (eV)**	**Relative E (eV)**
**STEP 1**	Reactants	0.00 (0.00)	0.00 (0.00)	0.00 (0.00)
	E_b of cis-formic acid	-0.24 (-0.31)	-0.22 (0.29)	-0.16 (-0.23)
	TS for H_2 loss	-0.06 (-0.09)	0.14 (0.10)	0.09 (0.06)
	H_2 loss	-0.95 (-1.04)	-0.71 (-0.86)	-0.76 (-0.89)
**STEP 2**	H_2 loss	0.00 (0.00)	0.00 (0.00)	0.00 (0.00)
	TS breaking Ag-O bond	1.70 (1.75)	1.67 (1.69)	1.69 (1.73)
	O-bound ligand	1.43 (1.37)	1.22 (1.20)	1.30 (1.27)
	TS for CO_2 loss	1.86 (1.80)	1.65 (1.69)	1.64 (1.69)
	Formation of CO_2	0.81 (0.83)	0.60 (0.63)	0.67 (0.71)
	Separated products	0.95 (1.04)	0.71 (0.71)	0.76 (0.76)

Supplementary Table 4 | High-resolution mass spectrometry experiments comparing the mass-to-charge ratio (*m/z*) of the most abundant peak in the isotope pattern of a given ion. Experiments were recorded using an FT-ICR mass analyser from a Thermo LTQ-FT hybrid instrument. The theoretical *m/z* was determined from the molecular formula and charge state of the silver cluster using the Thermo Xcalibur Qual Browser version 2.2 software with the output style set to Profile (Gaussian) and the resolution set to 20000 and the valley set to full-width-half-mass (FWHM).

**Ion**	**Formula**	**Experimental (*m/z*)**	**Theoretical (*m/z*)**	**Error (ppm)**
**1a** $[Ag_2(H)]^+$	Ag_2H	216.81726	216.81713	0.6
$[Ag_2(H)(HO_2CH)]^+$	$Ag_2H_3C_1O_2$	262.82273	262.82262	0.4
$[Ag_2(H)(HO_2CH)_2]^+$	$Ag_2H_5C_2O_4$	308.82835	308.82811	0.8
**1b** $[(Ph_3P)Ag_2(H)]^+$	$Ag_2H_{16}C_{18}P$	478.90842	478.90833	0.2
$[(Ph_3P)Ag_2(H)(HO_2CH)]^+$	$Ag_2H_{18}C_{19}PO_2$	524.91391	524.91383	0.1
**1c** $[(Ph_3P)_2Ag_2(H)]^+$	$Ag_2H_{31}C_{36}P_2$	740.99997	740.99968	0.4
**1d** $[dppmAg_2(H)]^+$	$Ag_2H_{23}C_{25}P_2$	600.93701	600.93693	0.1
$[dppmAg_2(O_2CH)]^+$	$Ag_2H_{23}C_{26}P_2O_2$	644.92690	644.92678	0.2
**1e** $[dppbzAg_2(H)]^+$	$Ag_2H_{25}C_{30}P_2$	662.95277	662.95264	0.2
$[dppbzAg_2(O_2CH)]^+$	$Ag_2H_{25}C_{31}P_2O_2$	706.94262	706.94249	0.2
**1f** $[dppeAg_2(H)]^+$	$Ag_2H_{25}C_{26}P_2$	614.95271	614.95259	0.2
$[dppeAg_2(O_2CH)]^+$	$Ag_2H_{25}C_{27}P_2O_2$	658.94256	658.94245	0.1

Supplementary Methods

Chemicals used

Chemicals purchased from the following suppliers were used without further purification. (i) Ajax Finechem: formic acid (HCO_2H, 99%), silver(I) nitrate ($AgNO_3$),. (ii) Sigma-Aldrich: silver(I) tetrafluoroborate ($AgBF_4$, 98%), sodium formate (NaO_2CH, 99%) bis(dphenylphosphino)methane (dppm, 97%), 1,2-bis(diphenylphosphino)ethane (dppe, 99%), 1,2-bis(diphenylphosphino)benzene (dppb, 97%) (iii) Merck: acetonitrile (HPLC grade), methanol (AR grade) dichloromethane (AR grade). (iv) Riedel-de Haën: triphenylphosphine (PPh_3).

Gas-phase preparation of 1a-1f

All of the binuclear silver hydride cations, $[LAg_2(H)]^+$, were synthesized by CID of appropriate precursor complexes formed via electrospray ionization (ESI) of solutions containing silver(I) salts, a carboxylic acid or sodium formate and where required, the phosphine ligand. The solvent was found to play a key role in the formation of $[LAg_2(H)]^+$ via ESI/MS (Supplementary figure 1). In the case of triphenyl phosphine, $[(Ph_3P)_2Ag_2(O_2CH)]^+$, was only formed in a 1:1 methanol:dichloromethane mixture (Supplementary figure 1a). For the bisphosphine ligands, dppm gave the simplest ESI mass spectrum, with the base peak being $[dppmAg_2(O_2CH)]^+$ *m/z* 645, (Supplementary figure 1b). Modest signals of $[dppbAg_2(O_2CH)]^+$ *m/z* 707, (Supplementary figure 1d) and $[dppeAg_2(O_2CH)]^+$ *m/z* 659, (Supplementary figure 1e) were obtained, with other ions dominating the ESI mass spectra.

The hydride ligand is unmasked via fragmentation of a coordinated carboxylate ligand. Thus sequential CID of $[Ag_2(O_2C(CH_2)_2Ph)(MeCN)]^+$ (*m/z* 406, Supplementary figure 2a and 2b) gave **1a** (*m/z* 215), while $[(Ph_3P)_2Ag_2(O_2CH)]^+$ (*m/z* 785) underwent decarboxylation to give $[(Ph_3P)_2Ag_2(H)]^+$ (**1c** *m/z* 741, equation (1a)) in competition with loss of a phosphine ligand to give $[(Ph_3P)Ag_2(O_2CH)]^+$ (*m/z* 523, equation (2b), Supplementary figure 3a). The latter can be mass selected and

subjected to CID to yield $[(Ph_3P)Ag_2(H)]^+$ via decarboxylation (**1b** *m/z* 741, Supplementary equation (2), Supplementary figure 3b).

$[(Ph_3P)_2Ag_2(O_2CH)]^+$	→	$[(Ph_3P)_2Ag_2(H)]^+$	+	CO_2	(1a)
	→	$[(Ph_3P)Ag_2(O_2CH)]^+$	+	Ph_3P	(1b)
$[(Ph_3P)Ag_2(O_2CH)]^+$	→	$[(Ph_3P)Ag_2(H)]^+$	+	CO_2	(2)

CID of $[dppmAg_2(O_2CH)]^+$ *m/z* 645, results in the loss of CO_2 (equation (4), Supplementary figure 4a) yielding $[dppmAg_2(H)]^+$ *m/z* 601, **1d**. The other binuclear silver hydride cations, $[dppbzAg_2(H)]^+$, **1e** and $[dppeAg_2(H)]^+$, **1f** were formed in similar decarboxylation reactions (equation (4), Supplementary figure 4b and 4c), although the nature of the ligand influences both the relative ease of decarboxylation as determined via resolved CID, following the order $[dppeAg_2(O_2CH)]^+ \approx [dppbzAg_2(O_2CH)]^+ > [dppmAg_2(O_2CH)]^+$ (Supplementary figure 5). The energy resolved CID experiments show that $[dppbzAg]^+$ arises from secondary fragmentation of $[dppbzAg_2(H)]^+$ (Supplementary figure 6).

Energy-resolved collision-induced dissociation

Energy-resolved CID experiments were carried out using a Finnigan 3D ion trap (LCQ) mass spectrometer. The experimental method of Brodbelt was adapted[1], whereby the silver clusters were diluted to 50 μM and introduced into the mass spectrometer via a syringe pump set at 5 $\mu L.min^{-1}$ through a Finnigan ESI source. The source conditions used for optimum intensity of the target ions were: spray voltage 4.6 – 5.2 kV, capillary temperature 200°C, nitrogen sheath gas pressure, 40 (arbitrary units), capillary voltage 42 V, tube lens voltage -15 V. The mass-selected precursor ion was isolated with a mass selection window of 8 Th and an activation time of 10 ms. The normalised collision energy (NCE) was increased incrementally by 1.0 % typically starting from a NCE where no fragmentation is observed, until reaching the NCE required for depleting the precursor ion to < 5 % relative intensity. The NCE was converted to an amplitude of the resonance excitation RF voltage (tick amp),

Supplementary equation 3. The ‘tick amp slope” and “tick amp intercept” are taken from the latest calibration file prior to experiments. The Σproduct ions/total ion count (Y-axis) was plotted against the Activation Voltage determined from Supplementary equation 3.

$$\text{Activation Voltage (V)} = (\text{NCE})\% \div 30\% \, ((\text{parent mass})(\text{tick amp slope}) + \text{tick amp intercept}) \quad (3)$$

Relative ease of decarboxylation of 1d, 1e and 1f.

Method 1, Colorado and Brodbelt[1]:

The threshold activation voltage corresponding to the critical energy of decarboxylation was determined at 10% reaction extent, i.e. when Σproduct ions/total ion count = 0.1. The thresholds for dissociation were also measured for two systems were the critical energies for dissociation are known from other gas-phase experiments: $Ag(CH_3OH)^+$ (1.43 ± 0.16 eV)[2] and $Fe(C_5H_5)_2^+$ (3.7 ± 0.3 eV)[3]. The results of this plot are shown in Supplementary figure 5a and 5b. We find the following activation voltages ($V_{p\text{-}p}$): $[dppeAg_2(O_2CH)]^+$ (0.582) ≈ $[dppbzAg_2(O_2CH)]^+$ (0.585) < $[dppmAg_2(O_2CH)]^+$ (0.621).

Method 2, Falvo et al[4]:

The threshold activation voltage corresponding to the critical energy of decarboxylation for **1d**, **1e** and **1f** was determined by linear regression analysis of the relative abundance for the product of decarboxylation. The results of these plots are shown in Supplementary figure 5c – 5e. We find the following activation voltages ($V_{p\text{-}p}$): $[dppeAg_2(O_2CH)]^+$ (0.564) ≈ $[dppbzAg_2(O_2CH)]^+$ (0.566) < $[dppmAg_2(O_2CH)]^+$ (0.627).

Comparisons of experimental methods 1 and 2 to Theory:

The experimentally determined relative ease of decarboxylation via both the 10% reaction extent method of Brodbelt and the linear regression analysis method of Falvo are in agreement with the DFT calculated activation energies (eV) for decarboxylation of $[dppmAg_2(O_2CH)]^+$, $[dppbzAg_2(O_2CH)]^+$ and $[dppeAg_2(O_2CH)]^+$.

Supplementary References

(1) Colorado, A. & Brodbelt, J. An empirical approach to estimation of critical energies by using a quadrupole ion trap *J. Am. Soc. Mass Spectrom.*, **7**, 1116–1125 (1996).

(2) El Aribi, H.; Shoeib, T.; Ling, Y.; Rodriquez, C. F.; Hopkinson, A. C. & Siu, K. W. M. Binding Energies of the Silver Ion to Small Oxygen-Containing Ligands: Determination by Means of Density Functional Theory and Threshold Collision-Induced Dissociation *J. Phys. Chem. A* **106**, 2908–2914 (2002).

(3) Faulk, J. D. & Dunbar, R. C. Time-resolved photodissociation of gas-phase ferrocene cation: energetics of fragmentation and radiative relaxation rate at near-thermal energies *J. Am. Chem. Soc.* **114**, 8596–8600 (1992).

(4) Falvo, F.; Fiebig, F.; Dreiocker, F.; Wang, R.; Armentrout, P.B. & Schäfer, M. Fragmentation reactions of thiourea- and urea-compounds examined by tandem MS-, energy-resolved CID experiments, and theory, *Int. J. Mass Spectrom.*, **330–332**, 124–133 (2012).

(5) Halupka, M., Sander, W. A simple method for the matrix isolation of monomeric and dimeric carboxylic acids. Spectrochim. Acta A, **54**, 495–500 (1998).

(6) Su, T. & Bowers, M. T. Classical ion-molecule collision theory *Gas-Phase Ion Chemistry* **1**, 83–118 (1979).

(7) Lim, K. F. *Quantum Chemistry Program Exchange* **14**, 3 (1994). The program Colrate is available for download from the author's website at Deakin University, Geelong, Victoria, Australia: http://www.deakin.edu.au/~lim/programs/COLRATE.html.
